# Genetic analysis of resistance to powdery mildew on 7M^g^ chromosome of wheat–*Aegilops geniculata*, development and utilization of specific molecular markers

**DOI:** 10.1186/s12870-022-03934-w

**Published:** 2022-12-03

**Authors:** Yongfu Wang, Jianzhong Fan, Yi Xiao, Xianbo Feng, Hong Zhang, Chunhuan Chen, Wanquan Ji, Yajuan Wang

**Affiliations:** 1 College of Agronomy, Northwest A&F University, 712100 Yangling, China; 2 State Key Laboratory of Crop Stress Biology for Arid Areas, 712100 Yangling, China; 3 Shaanxi Research Station of Crop Gene Resources and Germplasm Enhancement, Ministry of Agriculture, 712100 Yangling, China

**Keywords:** *Aegil*ops *geniculata* Roth, Genetic analysis, Powdery mildew, Molecular cytogenetics, SLAF-seq

## Abstract

**Background:**

Powdery mildew caused by *Blumeria graminis* f. sp. *tritici* (*Bgt*) is prevalent in the main wheat-producing regions of China, resulting in severe yield losses in recent years. Mining and utilization of resistant genes from wild relatives of wheat is the most environmentally sound measure to control disease. *Aegilops geniculata* Roth (2n = 2x = 28, U^g^U^g^M^g^M^g^) is an essential and valuable disease-resistance gene donor for wheat improvement as a close relative species.

**Results:**

In this study, to validate powdery mildew resistance locus on chromosome 7M^g^, two genetic populations were constructed and through crossing wheat – *Ae. geniculata* 7M^g^ disomic addition line NA0973-5-4-1-2-9-1 and 7M^g^ (7 A) alien disomic substitution line W16998 with susceptible Yuanfeng175 (YF175, authorized varieties from Shaanxi province in 2005), respectively. Cytological examination, in situ hybridization (ISH), and functional molecular markers analysis revealed that the plants carrying chromosome 7M^g^ showed high resistance to powdery mildew in both F_1_ and F_2_ generation at the seedling stage. Besides, 84 specific markers were developed to identify the plants carrying chromosome 7M^g^ resistance based on the specific-locus amplified fragment sequencing (SLAF-seq) technique. Among them, four markers were selected randomly to check the reliability in F_2_ segregating populations derived from YF175/NA0973-5-4-1-2-9-1 and YF175/W16998. In summary, the above analysis confirmed that a dominant high powdery mildew resistance gene was located on chromosome 7M^g^ of *Ae. geniculata.*

**Conclusion:**

The results provide a basis for mapping the powdery mildew resistance gene mapping on chromosome 7M^g^ and specific markers for their utilization in the future.

**Supplementary Information:**

The online version contains supplementary material available at 10.1186/s12870-022-03934-w.

## Background

Wheat(*Triticum aestivum* L., 2n = 6x = 42, AABBDD)is the cultivated cereal crops with the broadest distribution, and the highest total output in the world, feeding 35 ~ 40% of the world population [1, 2]. However, with the change of climate and environment and the occurrence of diseases, the wheat productivity is seriously threatened [3–5]. Powdery mildew, caused by *Blumeria graminis* f. sp. *tritici* (*Bgt*), is one of the most harmful foliar diseases of wheat worldwide and can seriously lead to the loss of wheat yield once the epidemic occurs [6, 7]. Presently, a series of powdery mildew resistance genes, such as *Pm51*, *Pm55*, *Pm57*, *Pm58* and *Pm66* [8–12], have been identified from related species of wheat. Nonetheless, powdery mildew resistance is the frequent loss of effectiveness due to continuous pathogen mutations under extreme environmental changes. Therefore, mining and identifying more new powdery mildew resistance genes in wheat breeding is urgently needed [9–11, 13–17].

*Aegilops geniculata* Roth (2n = 2x = 28, U^g^U^g^M^g^M^g^) is an annual allotetraploid of *Aegilops* sect. *Aegilops*. Its chromosome configuration is derived from the diploids *Ae. comosa* Sm (2n = 2x = 14, MM) and *Ae. umbellulata* Zhuk. (2n = 2x = 14, UU), which is mainly distributed around the Mediterranean Sea, western and central Asia [18, 19], having excellent traits such as resistance to diseases and insect pests, salt and alkali, and strong adaptability in long-term crop natural selection. Thus, *Ae. geniculata* is an important source of wild resistance genes in wheat genetics and improvement [20, 21]. As relative plants of wheat, living in the natural environment without artificial selection, could survive in all kinds of terrible environmental conditions, and has a lot of excellent genes and traits which are not possessed or have been lost in common wheat. The introduction of elite genes from wheat relatives into common wheat is an important way to increase wheat genetic diversity, broaden the genetic basis of wheat breeding and improve wheat varieties [22–25]. For example, in 2002, Zeller et al. found the powdery mildew resistance gene *Pm29* in *Ae. geniculata* and confirmed that it was located on chromosome 7D [26]. In 2007, Kuraparthy et al. found a stripe rust resistance gene *Yr40* and a leaf rust resistance gene *Lr57* on chromosome 5M^g^ of *Ae. geniculata* [27].

Molecular markers accelerated the study of wheat and enhanced the efficiency of new varieties. However, the existing molecular markers could no longer meet the rule of quickly and accurately identifying the resistance genes of relative species inserted into wheat. Fortunately, the emergence of next-generation sequencing provides solid technical support for discovering wheat wild gene breeding [28, 29]. Specific-locus amplified fragment sequencing (SLAF-seq) can be efficiently used in the development of wheat molecular markers. Recently, the development of markers specific to the Wheat–*Thinopyrum ponticum* 1J^s^(1D) disomic substitution line with a maximum success rate of 52.98% using SLAF-seq [30] gave a successful example. However, to the best of our knowledge, there were few reports on the development of *Ae. geniculata* specific molecular markers based on SLAF-seq technology.

*Ae. geniculata* SY159 (accession Y359) showed immunity resistance to powdery mildew at both seedling and adult stages [31, 32]. To utilize the *Ae. geniculata* SY159 resistance genes in wheat, distant hybridization and chromosome manipulation between Chinese Spring and SY159 have been carried out since 2009s. A series of wheat-*Ae. geniculata*, including alien chromosome disomic addition line 7M^g^ [32], disomic addition line 3M^g^ [33] and disomic substitution line 7M^g^(7 A) [34] have been characterized by molecular cytogenetic methods. Among wheat*-Ae. geniculata* derivative lines, alien chromosome 7M^g^ addition line NA0973-5-4-1-2-9-1 and 7M^g^ (7 A) substitution line W16998 exhibited high level of resistances to powdery mildew in wheat-growing regions. The common wheat YF175 has good agronomic traits, but was highly susceptibility to powdery mildew. In this study, two genetic populations were constructed and through crossing NA0973-5-4-1-2-9-1 and W16998 with YF175, respectively. The objectives of the present study were to (1) construct and genetic analyze chromosome 7M^g^ by two genetic populations; (2) track the resistances of chromosome 7M^g^ using molecular cytogenetic and functional molecular markers methods; (3) develop and utilize more specific markers to verify the plants carrying chromosome 7M^g^ resistance using SLAF-Seq technique. The results provide a basis for alien resistance gene mapping and the marker utilization in the future wheat breeding.

## Result

### Cytological analysis of genetic population

Indoor root tip cytological identification was performed on plant materials CS, SY159, NA0973-5-4-1-2-9-1, W16998, YF175, their constructed F_1_ plants and F_2_ population. Cytological examination revealed that the number of chromosomes in CS was 42, while the chromosome number of SY159, NA0973-5-4-1-2-9-1, W16998 and YF175 were 28, 44, 42 and 42, respectively. Among them, the number of F_1_ plant chromosomes constructed by NA0973-5-4-1-2-9-1 and YF175 was 43 chromosomes in F_2_ plants varied between 42, 43, 44. The chromosome number of the F_1_ plant constructed by W16998 and YF175 was 42, and the chromosome number of the F_2_ plants were 40, 41, 42, 43 and 44 (Table 1). Thus, the results indicated that the F_2_ population produces different types of plants, which is great significance for selecting resistant materials.

**Table 1:** Statistics data for molecular cytological and disease resistance identification in target materials

materials	No. of plants	No. of observed	No. of	Identification of resistance	No. of plants present 7M^g^
		**chromosomes**	**cells**	**to powdery mildew**	**special SLAF-Seq markers**
CS	10	42	10	S	0
SY159	10	28	10	R	84
YF175	10	42	10	S	0
NA0973-5-4-1-2-9-1	10	44	10	R	84
F_1_ (YF175/NA0973-5-4-1-2-9-1)	10	43	10	S	84
F_2_ (YF175/NA0973-5-4-1-2-9-1)	165	42	84	S	0
		43	70	R	84
		44	60	R	84
W16998	10	42	10	R	84
F_1_ (YF175/W16998)	10	41	10	R	84
F_2_ (YF175/W16998)	143	40	100	S	0
		41	100	R	84
		41	100	S	0
		42	100	R	84
		42	100	R	84
		42	100	S	0
		43	100	R	84
		43	100	R	84
		44	100	R	84

*R* Resistant, *S* Susceptible

### FISH and FISH-GISH analysis

**Fig. 1:**
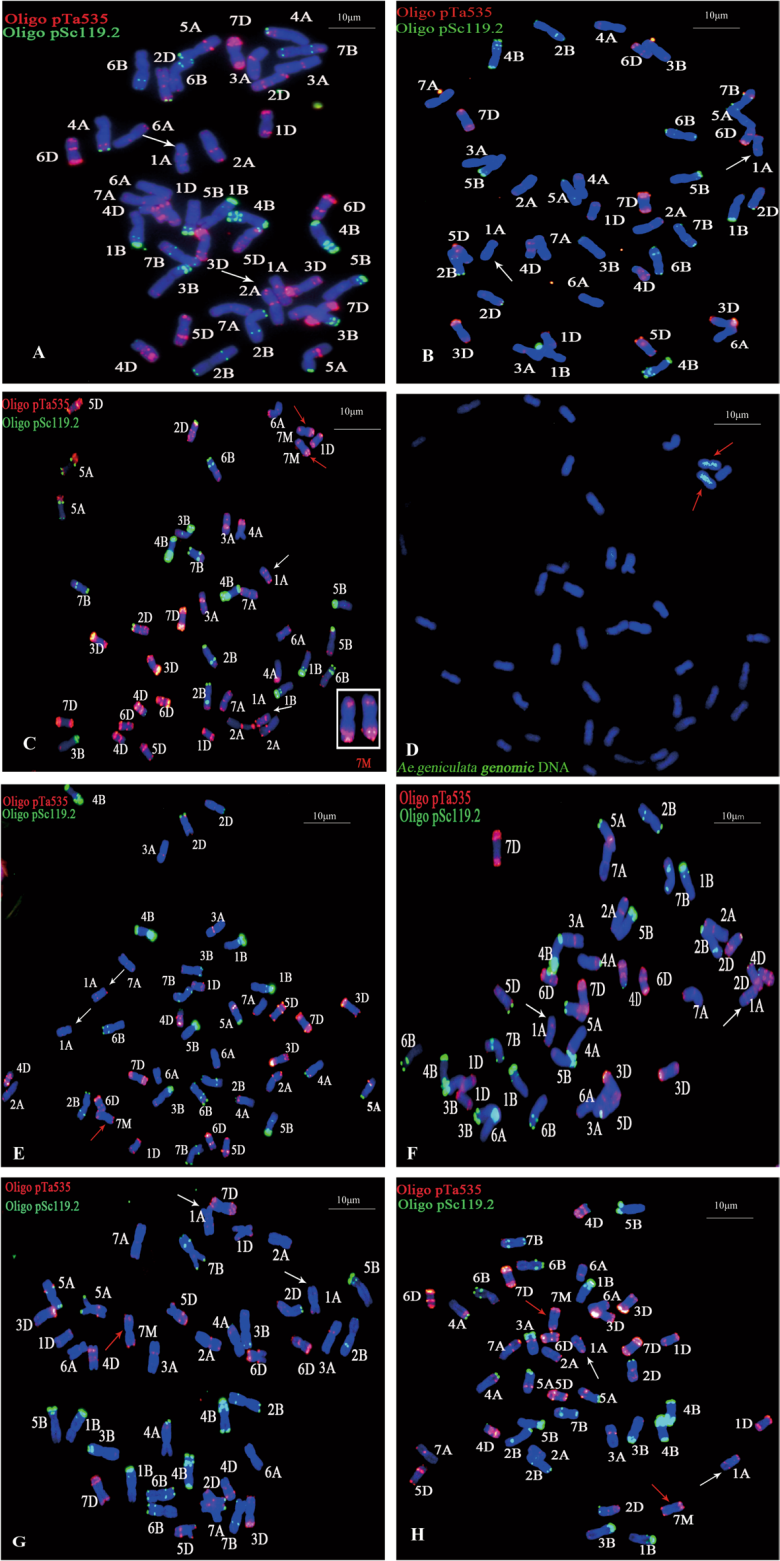
The chromosome karyotype of the target plant was analyzed by FISH and continuous GISH. FISH uses Oligo-pSc119.2 (green) and Oligo-pTa535 (red) as probes. SY159 genomic DNA was mixed with CS and used as a probe for sequence GISH. The red arrow indicates the alien chromosome introduced from SY159. The white arrow indicates chromosome 1 A. The structural variation of the chromosome was indicated by the white arrow chromosome. **A** FISH of CS. **B** Analysis of YF175 by FISH. **C** FISH of NA0973-5-4-1-2-9-1. **D** GISH of NA0973-5-4-1-2-9-1 in the same cell. **E**-**H** YF175 and NA0973-5-4-1-2-9-1constructed F_1_, F_2_ FISH. **E** F_1_ plant (2n = 43, adding one alien chromosome). **F** F_2_ plant (2n = 42, without alien chromosome). **G **F_2_ plant (2n = 43, adding one alien chromosome). **H** F_2_ plant (2n = 44, adding two alien chromosomes). Chromosomes were counterstained by DAPI (blue). The bar indicates 10 μm

**Fig. 2:**
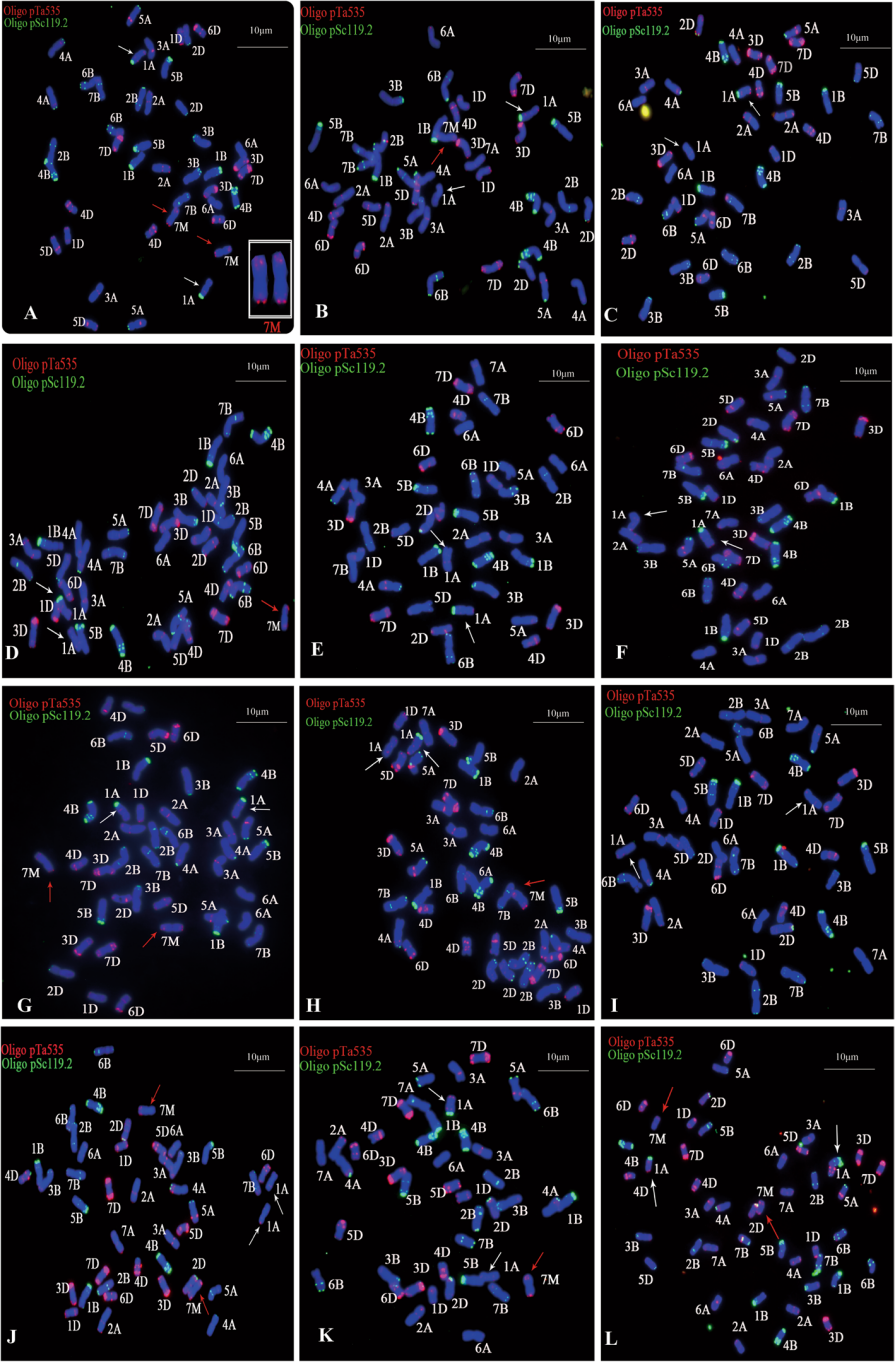
The karyotype of the target plant was analyzed by FISH. FISH uses Oligo-pSc119.2 (green) and Oligo-pTa535 (red) as probes. The red arrow indicates the alien chromosome introduced from SY159. The white arrow indicates chromosome 1 A. The structural variation of the chromosome was indicated by the white arrow chromosome. **A** FISH of W16998. **B** FISH of F_1_. (C-L) F_2_ constructed by YF175 and W16998. **C** F_2_ plant (2n = 40, missing two chromosome 7 A). **D** F_2_ plant (2n = 41, with one alien chromosome and missing two 7 A). **E** F_2_ plant (2n = 41, missing one chromosome 7 A). **F** F_2_ plant (2n = 41, missing one chromosome 7 A). **G** F_2_ plant (2n = 42, two alien chromosomes replaced two 7 A). **H** F_2_ plant (2n = 42, one alien chromosome replaced one 7 A). **I** F_2_ plant (2n = 42, without alien chromosome). **J** F_2_ plant (2n = 43, adding two alien chromosomes and missing one 7 A). **K** F_2_ plant (2n = 43, adding one alien chromosome). **L** F_2_ plant (2n = 44, adding two alien chromosomes). Chromosomes were counterstained by DAPI (blue). The bar indicates 10 μm

The chromosomes of the target materials were analyzed by the FISH with oligonucleotide probes Oligo-pTa535 and Oligo-pSc119.2, and the chromosomes were compared with CS [35] standard karyotype map (Fig. 1). Comparing FISH karyotype map of CS (Fig. 1A), YF175 (Fig. 1B) and NA0973-5-4-1-2-9-1 (Fig. 1C), NA0973-5-4-1-2-9-1 have 44 chromosomes and a similar FISH bind variation apart from two alien chromosomes (Fig. 1). F_1_ plant of cross YF175/NA0973-5-4-1-2-9-1 has 43 chromosomes containing one alien chromosome (Fig. 1E). In F_2_ plants, the chromosomes numbers varying among 42, 43 and 44 were formed. Of which, the number of alien chromosomes was zero, one and two, respectively (Fig. 1 F-H) (Additional file: Figure S1. Uncropped images of Fig. 1). The results of FISH showed that there also have two 7M^g^ chromosomes in W16998 but missing 7 A (Fig. 2 A). Meanwhile, there was a variation of chromosome 1 A in Fig. 2A. Chromosome 7M^g^/7A heterozygote could be stably found in F_1_ plants of the cross YF175/W16998 (Fig. 2B). Screening the FISH map of F_2_ plants derived from YF175/W16998 the chromosome number was significantly varied, including 40 (containing zero alien chromosome), 41 (including zero or one alien chromosome), 42 (having zero, one or two alien chromosomes), 43 (containing one or two alien chromosomes) and 44 (including two alien chromosomes) (Fig. 2C-L). In summary, Figs. 1 and 2 showed that alien chromosomes could be expectedly inherited and produce different gamete types, which was of great significance in wheat breeding (Additional file: Figure S2. Uncropped images of Fig. 2).

### Identification of powdery mildew resistance

**Fig. 3:**
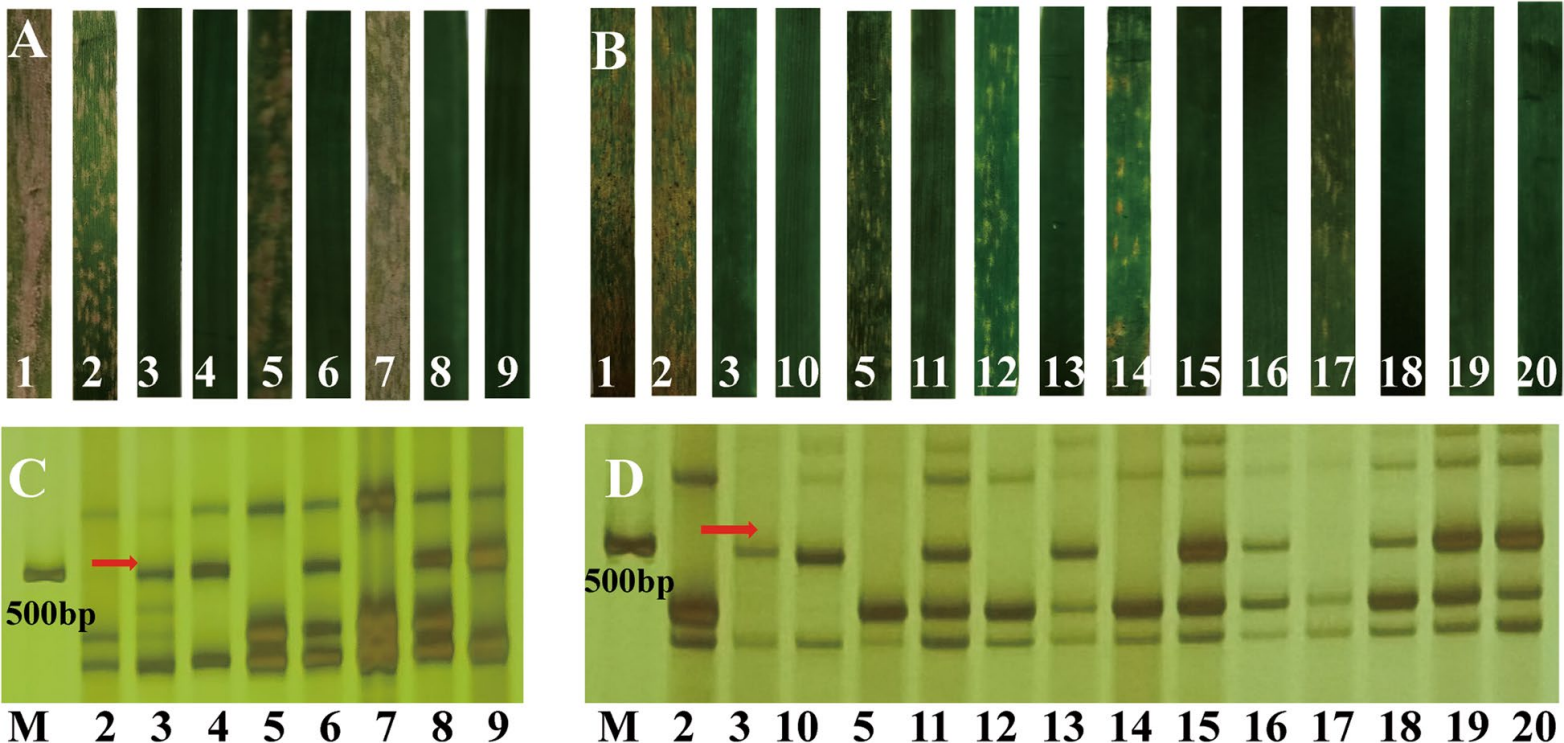
Powdery mildew reaction (**A-B**) and EST–STS functional molecular marker analysis of the target materials (**C-D**). (1) SY225. (2) CS. (3) SY159. (4) NA0973-5-4-1-2-9-1. (5) YF175. (6) YF175 × NA0973-5-4-1-2-9-1 F_1_ plant. (7–9) Three genotypes in YF175 NA0973-5-4-1-2-9-1 F_2_ population. (7) F_2_ plant (2n = 42, without alien chromosome). (8) F_2_ plant (2n = 43, adding one alien chromosome). (9) F_2_ plant (2n = 44, adding two alien chromosomes). (10) W16998. (11) YF175 x W16998 F_1_ plant. (12–20) Nine genotypes in YF175 x W16998 F_2_ population, (12) F_2_ plant (2n = 40, missing two chromosome 7 A). (13) F_2_ plant (2n = 41, adding one alien chromosome and missing two 7 A). (14) F_2_ plant (2n = 41, missing one chromosome 7 A). (15) F_2_ plant (2n = 42, two alien chromosomes replaced two 7 A). (16) F_2_ plant (2n = 42, one alien chromosome replaced one 7 A). (17) F_2_ plant (2n = 42, without alien chromosome). (18) F_2_ plant (2n = 43, adding two alien chromosomes and missing one 7 A). (19) F_2_ plant (2n = 43, adding one alien chromosome). (20) F_2_ plant (2n = 44, adding two alien chromosomes). (M) DL2000 (2 kb DNA ladder). **C**-**D** The EST–STS markers amplification results with *BE637663*. The red arrows state the specific bands

After the plants in A and B pots were inoculated with powdery mildew E09 and the resistance to powdery mildew was identified when they were fully infected. The results demonstrated that CS, YF175 and susceptible control Shaanyou 225 (SY225) exhibited spore development at the four grades level, while SY159 and its’ derivation lines, NA0973-5-4-1-2-9-1 and W16998 showed grade 0 and immunity resistance to *Bgt* E09. The F_1_ plants of YF175/NA0973-5-4-1-2-9-1 and YF175/W16998 showed high resistance. Among 165 F_2_ plants of YF175/NA0973-5-4-1-2-9-1, 122 plants showed resistant and 43 showed susceptible to *Bgt* E09, fitted to a ratio of 3:1 approximately (χ^2^_3:1_ = 0.06388, *P* = 0.8005) (Fig. 3A; Table 1). Notably, all resistant plants have 43 (adding one alien chromosome) and 44 chromosomes (adding two alien chromosomes), whereas all plants containing 42 chromosomes (without alien chromosome) showed susceptibility to *Bgt* E09. Similarly, the F_1_ plant of YF175/W16998 also showed high resistance to *Bgt* E09 at the seedling stage. Nine genotypes could be detected in YF175/W16998 derived F_2_ population, although the chromosome number ranged from 40 to 44. The detailed chromosomes configuration could be classed into as follow: 40 (missing two chromosome 7 A), 41 (with one alien chromosome and missing two chromosome 7 A), 41 (missing one chromosome 7 A), 42 (two alien chromosome replaced two 7 A), 42 (one alien chromosome replaced one 7 A), 42 (without alien chromosome) 43 (adding two alien chromosomes and miss one 7 A), 43 (add one alien chromosome), and 44 (adding two alien chromosomes) chromosomes respectively. Among 143 F_2_ plants, the segregation of resistant Vs susceptible plants is 100: 43 plants, approximated a 3:1 ratio (χ^2^_3:1_ = 0.8570, *P* = 0.3546) (Fig. 3B; Table 1). FISH tested results showed that all resistant plants carried alien chromosome 7M^g^. To sum up, genetic analysis preliminarily showed that only the materials carrying chromosome 7M^g^ were resistant. The chromosome carried a dominant gene for powdery mildew resistance, which could be stably inherited in the population.

### Functional molecular marker analysis

To determine the homologous group relationship of the target materials, DNA was extracted from fresh leaves of the target materials by a modified CTAB method [36]. Then, a previously developed EST-STS molecular marker *BE637663* of chromosome 7M^g^ [32] was used to analyze resistant-related bands CS, SY159, NA0973-5-4-1-2-9-1, W16998, YF175, F_1_ and F_2_ plants. The results showed that the primer *BE637663* (Table 2) could amplify chromosome-specific bands and correspond to disease resistance (specific bands appeared in disease-resistant materials whereas no specificity appeared in susceptible materials) (Fig. 3C-D). It was further proved that the reported disease-resistance gene resided in chromosome 7M^g^. (Additional file: Figure S3. Uncropped gel images of Fig. 3).

### Specific-locus amplified fragment sequencing analysis

**Fig. 4:**
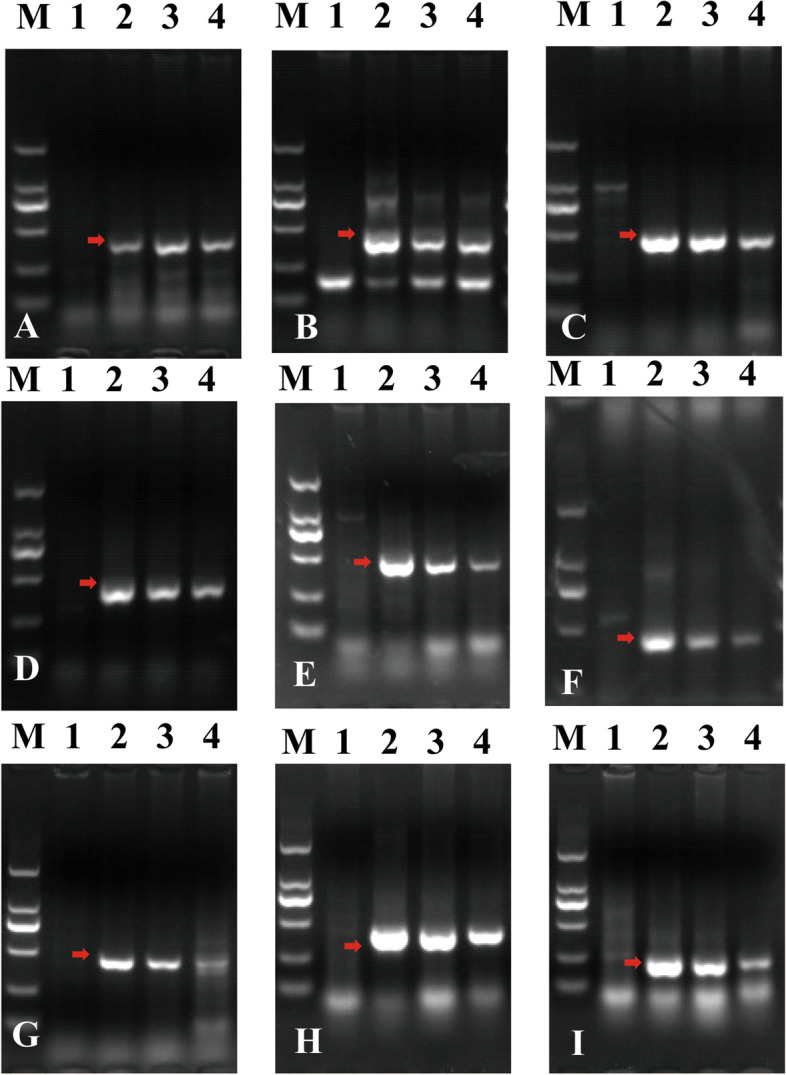
Amplification patterns of *Aegilops* accessions using NA0973-5-4-1-2-9-1 and W16998. **A-I** SLAF markers *Marker7*, *Marker9*, *Marker65*, *Marker945*, *Marker1661*, *Marker2024*, *Marker887*, Marker2104 and Marker390. (M) DL2000 (2 kb DNA ladder). (1) CS. (2) SY159. (3) NA0973-5-4-1-2-9-1. (4) W16998. The red arrows indicate the specific bands

To develop new molecular markers for this resistance loci, we generated many sequences data through SLAF sequencing. The SLAF-seq is data comprised of a total of 4,733,453, 8,752,434, 8,627,924 and 6,977,175 clean reads for CS, SY159, NA0973-5-4-1-2-9-1 and W16998, respectively. The average Q30 of sequencing was 94.15%, and the average GC content was 48.66%. After filtering out low-depth data, the final numbers of SLAF-seq reads (reads size 150 bp) were 314,571, 193,701, 388,039 and 362,377 for CS, SY159, NA0973-5-4-1-2-9-1 and W16998, respectively, and the average sequencing depth of the tags was 74.11x. Using the Burrows-Wheeler Alignment (BWA) tool, 2391 reads in NA0973-5-4-1-2-9-1 showed 50% similarity to the CS wheat reference genome (IWGSC-RefSeqv1.0). Among them, there were 961 reads with more than 90% similarity to SY159. In addition, it was found that the similarity of 3980 reads between W16998 and CS was more than 50%. Among them, there were 782 reads with more than 90% similarity to that in SY159. Finally, the reserved reads of NA0973-5-4-1-2-9-1 and W16998 were compared and analyzed, and then the 339 overlapped reads were considered to be specific fragments of chromosomes 7M^g^. According to sequence information, 121 primers were designed to screen and identify CS, SY159, NA0973-5-4-1-2-9-1, and W16998 (Table 2, Additional file: Table S2). Among them, 84 markers showed specific bands indicating chromosome 7M^g^, with a success rate of 69.4% (Fig. 4; Table 1). This means that 84 sequences of chromosome 7M^g^ could be used as specific probes for identifying alien genetic material of *Ae. geniculata* (Additional file: Figure. S4. Uncropped gel images of Fig. 4). This provides technical support for utilizing excellent genes on alien chromosomes.

**Table 2:** Expressed sequence tag-sequence-tagged site (EST–STS) and special SLAF-seq markers list 7M^g^

Marker	Type	Primer (5’-3’)	Location	Geltype/Restrictionenzyme	Tm ◦C/t (h)
*BE637663*	EST-SSR	F: ACTGTTGCTTCGCTCCAAGT	7AL 7BL 7DL	8% non-denaturing	60
		R: GTTCCATTTCCGATGTGCTC		polyacrylamide gel-	
*Marker7*	SLAF-seq	F: GCTACACCGAACGACAATCA	7M^g^	2% agarose gel	58
		R: AGGTGTCCGCTAAGGATTGA			
*Marker9*	SLAF-seq	F: CAGACGAGCTTGACTGCTTG	7M^g^	2% agarose gel	58
		R: AATTTGTGCCGATTCAAAGG			
*Marker65*	SLAF-seq	F: GCTGCAGCAAAGCATTTACA	7M^g^	2% agarose gel	58
		R: AGCACGTCCAAAAGAGGCTA			
*Marker945*	SLAF-seq	F: CTTCCGAGCTCCGGTAAGAT	7M^g^	2% agarose gel	58
		R: TGATGTGGTGATCCTTTCCA			
*Marker1661*	SLAF-seq	F: TTGGCATACAAGCCAACTCA	7M^g^	2% agarose gel	58
		R: AGAGTGCTCTGGTCGGAAAG			
*Marker2024*	SLAF-seq	F: CTTTGGTCCCTTCTTTGGGT	7M^g^	2% agarose gel	58
		R: GCTTAATAGATGCCGAAGCG			
*Marker887*	SLAF-seq	F: GCATGGGTCCTGTCGACTAT	7M^g^	2% agarose gel	58
		R: TCAGTGGCTGAAGGTCAGTG			
*Marker2104*	SLAF-seq	F: GCCCAAGGATGAGATGCTAA	7M^g^	2% agarose gel	58
		R: AGAAGAGGGGAAACTCGACC			
*Marker390*	SLAF-seq	F: GGAAGTTTACCGAAGATGGC	7M^g^	2% agarose gel	58
		R: GGGAGCTAAAGAAAGCCGAT			

**Fig. 5:**
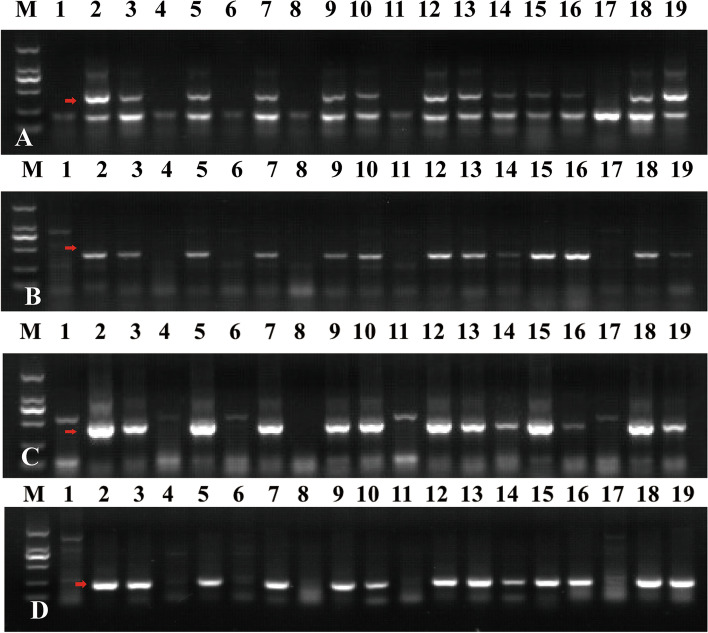
Molecular marker development and PCR amplification in the target materials. (M) DL2000 (2 kb DNA ladder (1) CS; (2) SY159; (3) W16998; (4) YF175; (5)YF175 x NA0973-5-4-1-2-9-1 F_1_ plant; (6–14) Nine genotypes in YF175 x W16998 F_2_ population; (6) F_2_ plant (2n = 40, missing two chromosome 7 A); (7) F_2_ plant (2n = 41, adding one alien chromosome and missing two 7 A); (8) F_2_ plant (2n = 41, missing one chromosome 7 A); (9) F_2_ plant (2n = 42, two alien chromosome replaced two 7 A); (10) F_2_ plant (2n = 42, one alien chromosome replaced one 7 A); (11) F_2_ plant (2n = 42, without alien chromosome); (12) F_2_ plant (2n = 43, adding two alien chromosomes and missing one chromosome 7 A); (13) F_2_ plant (2n = 43, adding one alien chromosome); (14) F_2_ plant (2n = 44, adding two alien chromosomes); (15) NA0973-5-4-1-2-9-1; (16)YF175 x NA0973-5-4-1-2-9-1 F_1_ plant; (17–19) Three genotypes in YF175 x NA0973-5-4-1-2-9-1 F_2_ population; (17) F_2_ plant (2n = 42, without alien chromosome); (18) F_2_ plant (2n = 43, adding one alien chromosome); (19) F_2_ plant (2n = 44, adding two alien chromosomes); **A**-**D** The SLAF-seq markers amplification results with *Marker9*, *Marker1661*, *Marker2024*, *Marker390*. The red arrows indicate the 7M^g^ specific bands

To further verify the reliability of the developed markers, *Marker9*, *Marker1661*, *Marker2024* and *Marker390* were randomly selected from the newly developed target chromosome SLAF-seq markers. PCR amplification analysis in CS, SY159, W16998, YF175, NA0973-5-4-1-2-9-1, F_1_ plants and F_2_ population (Fig. 5). *Marker9*, *Marker1661*, *Marker2024* and *Marker390* produced 420 (Fig. 5 A), 453 (Fig. 5B), 458 (Fig. 5C) and 228 bp (Fig. 5D) specific bands in all tested resistant plants, respectively, but could not in susceptible plants. Considering that the resistance to powdery mildew was linked with 7M^g^, as aforementioned, we could conclude that these specific markers could be efficiently used to distinguish chromosome 7M^g^ with a novel powdery mildew resistance gene. These specific molecular markers could help us further study and open opportunities to develop genomic resources for wild wheat to facilitate crop improvement (Additional file: Figure S5. Uncropped gel images of Fig. 5).

## Discussion

With the continuous development of breeding, the newly bred varieties were focused on better performance in yield and quality, which made the proportion of a few resistance genes higher and higher. For producing new varieties, although some genes that are not conducive to yield have been eliminated, beneficial genes have also been eliminated invisibly, resulting in a narrower genetic base of wheat itself [7, 37, 38]. Due to the loss of resistance genes in wheat, the discovery of new resistance genes from related wild species of wheat has also become one of concerns for breeders. Some resistance genes of wheat powdery mildew were derived from its wild-relatives [39]. The wild related genera of wheat reproduce under natural conditions and preserve a large number of original genes, which provides a large number of genetic resources for improving wheat genetics and breeding. Mining excellent genes from wild related species of wheat were of great significance in broadening wheat genetic resources, enriching the genetic diversity of wheat varieties, and promoting the breeding of new varieties [40]. Wheat distant hybridization is a long-standing and increasingly important method to improve the genetic diversity of common wheat by introducing beneficial genes from related species of grain [22, 41]. Combining distant hybridization and conventional breeding, Wang et al. have introduced *Fhb7* into diverse wheat backgrounds for wheat *Fusarium* head blight resistance improvement [42].

*Ae. geniculata* contains many excellent genes distributed on different chromosomes [31]. It has been a hot spot in wheat distant hybridization research to transfer its’ wonderful traits shown by these genes, because *Aegilops* and *Triticum* are most closely related with a substantial genetic similarity [43]. Previous studies have shown that four disease resistance-genes (*Pm29*, *Yr40*, *Lr57*, *Sr53*) were found in *Ae. geniculata* [26, 27, 44].

In this study, cytological identification and genetic analysis showed that a novel powdery mildew resistance gene was transferred into two new wheat derivation lines NA0973-5-4-1-2-9-1 and W16998. Interestingly, we observed the various type of chromosomes by constructing the F_1_ plant and F_2_ population, which lading a more abundant material foundation for the utilization of their excellent trait genes (Table 1). It is no doubt that this will be help in broadening wheat genetic resources, and enriching resistant genetic diversity in the future. FISH has become a valuable tool for detecting exogenous chromosomal sources [28]. Tang et al. developed some oligonucleotide probes based on new tandem repeat sequences, which were of great significance for identifying wheat chromosomes or specific chromosome segments [35]. The GISH technology described by Yang et al. was used with minor modifications [45]. In this study, we employed FISH and GISH techniques and determined that NA0973-5-4-1-2-9-1 and W16998 contained the same pair of alien chromosomes 7M^g^ (Figs. 1 and 2). However, comparing the karyotypes of individual plants in the two F_2_ populations through crossing with YF175, there have more classes of karyotypes in YF175/W16998. Nine karyotypes were found in their derivations (Fig. 2C-L), which laid a foundation for creating small fragment translocation lines and introgression lines. The tested results of powdery mildew showed that the response of plants containing chromosomes 7M^g^ components of SY159 to *Bgt* race E09 was consistent with that of parents NA0973-5-4-1-2-9-1 and W16998. In previous studies, a variation in the 1 A FISH pattern also existed in W16998 (Fig. 2A) of Wheat–*Ae. geniculata* 7M^g^ disomic substitution line was obtained, so the specific source of the powdery mildew resistance gene could not be clearly understood [34]. Fortunately, compared with the standard CS karyotype, we found that 1 A had no variation in Fig. 1, while the green signal appeared in the short arm of 1 A in Fig. 2. Besides, the ratio of both resistance Vs. susceptible plants and 7M^g^ with Vs. without in F_2_ population conformed to Mendelian genetic law but have not related to variation in chromosome 1 A. This fully suggested that the disease resistance was only associated with alien chromosome 7M^g^, and the population was highly resistant to powdery mildew whether it contained one or two exogenous bands (Fig. 3). This indicated that the powdery mildew resistance of chromosome 7M^g^ is stable and dominant inherited.

EST-SSR is an accurate and reliable way to identify the homologous relationship between alien chromosomes and wheat chromosomes. Wang et al. successfully identified rye 1RS chromosomes by EST-SSR markers [46]. At present, rye genetic maps have been successfully constructed by using different marker techniques [40]. Unfortunately, few markers were reported on *Ae. geniculata* since it was difficult to develop chromosome-specific markers for related species of wheat by conventional methods. For example, Wu et al. screened two RAPD markers specific to *Agropyron cristatum* from 520 RAPD primers [47]. In this study, we used a high-throughput sequencing technique for screening specific length DNA fragments by constructing a SLAF-seq library, and obtaining a large number of sequences by high-throughput sequencing technology, analyzing and comparing data with software, obtaining a large amount of sequence information. Furthermore, specific molecular markers were developed, as well as their specialization of sequences were evidenced. The effective rate of marker development reaches 69.4% here, which is similar to the efficiency of 65.9% in *Th. elongatum* [48], but higher than 40.33% in *Ae. biuncialis* [49] and previously reported 47.66% in *Ae. geniculata* [33]. This means that the SLAF technology was an economically effective method to develop specific markers from wheat-alien chromosome lines. Addition, the improvement of wheat genetics and breeding, compared with additional lines and substitution lines, breeders prefer translocation lines because they carry fewer unfavorable fragments and could be directly used in the improvement of wheat genetics and breeding [13, 50, 51]. To develop small-segment translocation or introgression lines with resistance genes, mutagenicity methodology is usually adopted to induce target chromosome variation, such as ^60^Coγ radiation [52]. This laboratory has obtained a number of high-generation irradiated materials containing the alien chromosome 7M^g^. In the later stage, the developed specific markers in this study could be not only used to quickly trace the existence of alien chromosomes under the background of wheat, but also to fine map the genes that control excellent traits on chromosome7M^g^. In a word, these results set a robust foundation for introducing the genetic materials of 7M^g^ into wheat in the future.

## Conclusion

In summary, here we evidenced that the powdery mildew resistance gene in chromosome 7M^g^ was a dominant gene with solid resistance. This available genetic resources in future wheat breeding. Moreover, a batch of markers that could detect the genetic materials of chromosome 7M^g^ of *Ae. geniculata* were developed using the tag sequence obtained by simplified genome sequencing for the first time. This laid a foundation for the further creation of translocation lines or introgression lines containing powdery mildew resistance genes.

## Materials and methods

### Plant materials

The wheat – *Ae. geniculata* 7M^g^ disomic addition line NA0973-5-4-1-2-9-1 and 7M^g^ (7 A) alien disomic substitution line W16998 were identified by Wang et al. [32, 34]. NA0973-5-4-1-2-9-1 and W16998 were derived from a cross between CS and *Ae. geniculata* SY159. They were crossed with powdery mildew susceptible to common wheat Yuanfeng175 (YF175), respectively. Then, the F_1_ and F_2_ generations were constructed strictly self-crossed, respectively. Common wheat Shaanyou 225 (SY225) was used as the susceptible control to assess powdery mildew resistance. The *Aegilops geniculata* SY159 (Unit preservation number Y359) was collected in Syria in 1992, and here kindly provided by Professor Lihui Li and Xinming Yang (the Institute of Crop Sciences, Chinese Academy of Agricultural Sciences. Beijing, 100,081, P. R. China). The common wheat YF175 (authorized varieties from Shaanxi province in 2005; accession 2,005,006) is high-quality strong gluten wheat with many excellent traits, but increased susceptibility to powdery mildew; common wheat SY225 was an authorized variety from Shaanxi province in 1993 (accession 1,993,257); common wheat ‘Chinese Spring’ (CS) (AABBDD, 2n = 42) is a local variety originating from Chengdu, Sichuan, China. All the materials were maintained at the Wheat Distant Hybridization and Molecular Chromosome Engineering Laboratory, the College of Agronomy, Northwest A&F University. Yangling, 712,100, P. R. China.

### Cytological analysis of genetic population

The target seeds were soaked in a petri dish with filter paper (the water in the dish should cover the seeds) and placed in a 23 °C incubator in the dark for about 24 h. Then, the water was poured from the petri dish, arranged neatly, and continued to put in the incubator, while making sure the petri dish remained moist throughout the rooting process. Following the method of Yang et al. [53], when the roots grew to 2–3 cm, the roots were excised and placed in a previously perforated centrifuge tube and treated with nitrous oxide (N_2_O) for ~ 2 h. Then, it was immobilized in 95% acetic acid for 30 min and stored in 70% ethanol for later use. The root tip was treated with 1% pectinase and 2% cellulose in a water bath at 37 °C for ~ 1 h, and then made into white tablets. At last, the chromosomes number of root tip cells were observed by an Olympus BX43 Microscope (Japan) equipped with a Photometrics SenSys CCD camera and recorded by photography.

### FISH and FISH-GISH analysis

The Oligonucleotide probes Oligo-pTa535(red) and Oligonucleotide probes Oligo-pSc119.2 (green) were used as fluorescence in situ hybridization, and compared with existing CS karyotypes to determine the alien chromosome profile [35]. Then, the white tablet after fluorescence in situ hybridization was soaked and dried in alcohol and exposed to dry, and then genomic in situ hybridization (GISH) was carried out. GISH would extract and purify SY159 DNA as a probe, and different concentrations of Chinese spring DNA would be mixed after blocking, according to the ratio of 1:300, at a suitable temperature of 56 °C for hybridization. Finally, the chromosome signals were observed under a phase contrast microscope to determine whether there was a foreign source. Combining the results of these two techniques could further determine whether the chromosomes of the derived materials carry alien genes. The chromosomes were stained with the blue-fluorescent DNA stain 4′,6-diamidino-2-phenylindole (DAPI) [32]. Select and analyze the karyotype image with better signal display (Olympus Bx-53).

### Assessment of powdery mildew resistance

The resistance to powdery mildew was assessed at the seedling stage in an artificial climate incubator. The populations constructed by NA0973-5-4-1-2-9-1 and W16998 were planted in pots A and B of 28 × 52 cm, respectively. In the A pot, the first to sixth rows planted SY225, CS, SY159, material NA0973-5-4-1-2-9-1, YF175, and F_1_ plants, respectively, with ten plants each row. A total of 165 F_2_ plants were planted in the remaining space. Similarly, in the B pot, SY225, CS, SY159, W16998 and YF175, and F_1_ plants were planted in the first to sixth rows, respectively. 143 F_2_ plants derived from YF175/W16998 were planted in the remaining space. All plants were inoculated with *Bgt* physiological race E09 by artificial shaking when conidiospores were obtained from pre-infected SY225 at the two-leaf stage. The powdery mildew phenotype was scored after SY225 was fully infected (∼2 weeks). According to the method stated in the study by Zhao et al. [54], I. e. the level of infection was divided into 0–4, 0 indicates immunity, 0; indicates near immunity, 1 indicates high resistance, 2 indicates moderate resistance, 3 indicates moderate sensitivity, and 4 indicates high sensitivity [55].

### Molecular marker analysis

According to the previous studies on the chromosomes of *Ae. geniculata* SY159 by Wang et al. [32, 34], which have selected only one EST-STS (Expressed sequence tag-sequence-tagged site) functional molecular marker *BE637663* from among the PCR Primers in Grain Genes database (http://wheat.pw.usda.gov/SNP/new/pcr _primers.shtml) [56] to study the two genetic populations. DNA amplification and later data processing were performed using the method described by Zhu et al. [57]. PCR products were subjected to polyacrylamide gel electrophoresis and stained with 1% AgNO_3_ solution. Finally, the different bands were observed and analyzed.

### Specific-locus amplified fragment sequencing analysis

In recent years, in the field of molecular markers, the development of specific heterogeneous chromosome markers has been of great significance in breeding. Then, SLAF-seq is a simplified genome sequencing technique that could be developed with specific molecular characteristics. SLAF-seq of DNA extracted from NA0973-5-4-1-2-9-1 and W16998 were performed with some modification by the Beijing Biomarker Technologies Corporation [48, 49]. To improve the specificity and efficiency of the marker, firstly, the SLAF sequences of NA0973-5-4-19-1 and W16998 were firstly compared with the CS sequences (http://www.wheatgenome.org/News/Latest-news/IWGSC-Reference-Sequencev1.0-browser-now-available-at-URGI), and the sequences with similarity less than 50% were reserved. Secondly, compared the preserved sequences with SY159 sequences, the sequences with more than 90% similarity were selected as candidate-specific sequences of *Ae. geniculata*. Finally, the overlapped sequence was used to design a specific primer [30, 33, 48]. The PCR amplification of modified was performed following the method previously described by Luan et al. [58]. The annealing temperature based on SLAF-seq markers were 59 ◦C. The amplified products were electrophoretic on a preprepared 2% agarose gel. After the end of electrophoresis, the polymorphism was observed and photographed on the gel imager. The specific primers designed in the experiment were synthesized by the Beijing Aoke Ding Sheng Biotechnology Co., Ltd. (Beijing, China).

## Supplementary Information

**Additional file 1.** 

## Data Availability

The datasets used and materials during the current study are available from the corresponding author on reasonable request. The data sets were deposited in the China National Center for Bioinformation (CNCB) database under accession number PRJCA009052.
