# Supplementary material for: Genetic analysis of resistance to powdery mildew on 7Mg chromosome of wheat–Aegilops geniculata, development and utilization of specific molecular markers
Source: BMC Plant Biol. 2022 Dec 3;22:564. doi: 10.1186/s12870-022-03934-w (PMC9719254; doi:10.1186/s12870-022-03934-w)
Supplement: Supplementary file 1 — Additional file 1. [file 12870_2022_3934_MOESM1_ESM.pdf]

Figure S1. Uncropped images of Fig. 1.

The chromosome karyotype of the target plant was analyzed by FISH and continuous GISH.

A

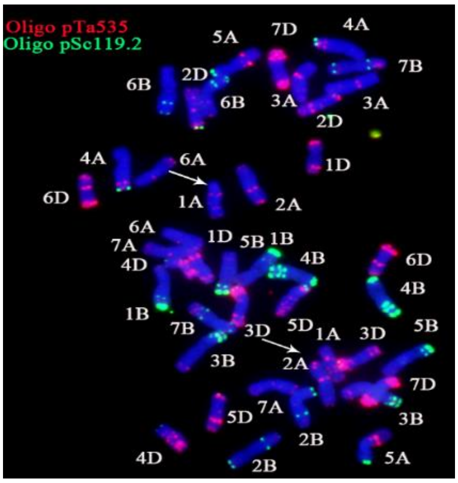

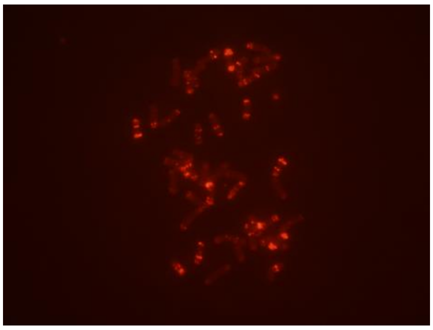

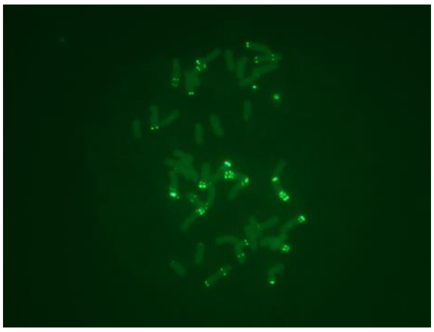

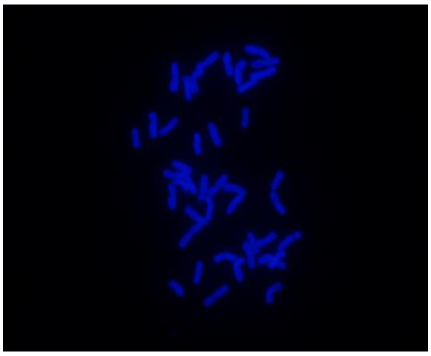

B

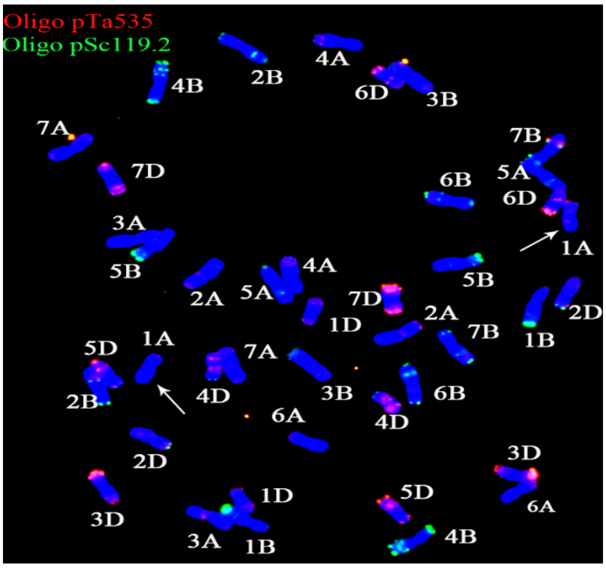
Oligo pTa535
Oligo pSc119.2
4B
2B
4A
6D
3B
7A
7D
7B
5A
6B
6D
1A
3A
5B
2A
5A
4A
7D
5B
1D
2A
2D
1B
7B
5D
1A
7A
2B
4D
3B
6B
4D
6A
2D
3D
6A
3D
1D
5D
3A
1B
4B

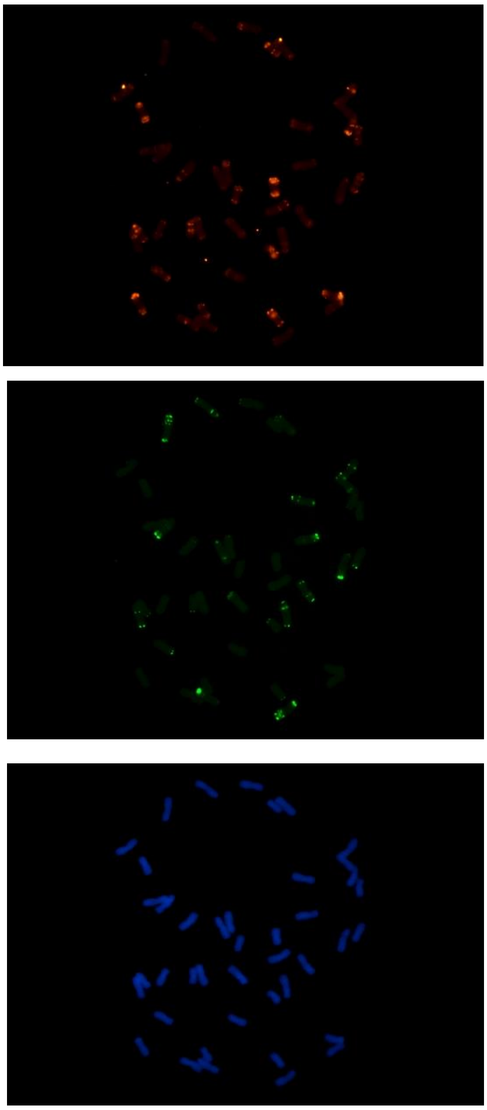

C

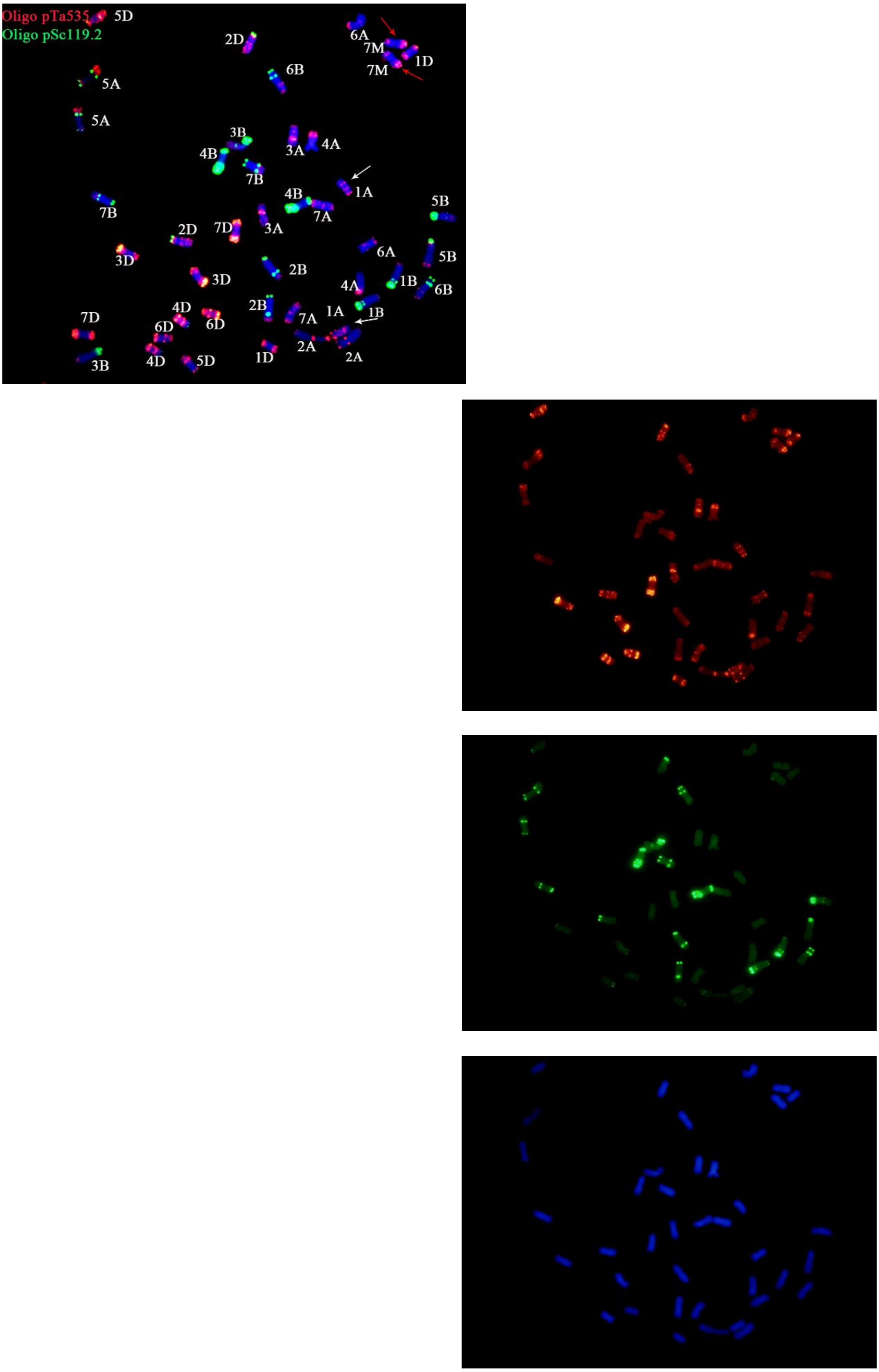

Oligo pTa535
Oligo pSc119.2
5D
2D
6A
7M
1D
7M
6B
5A
5A
3B
3A
4A
4B
7B
1A
4B
7A
5B
7B
2D
7D
3A
6A
3D
2B
5B
3D
4A
1B
6B
2B
7A
1A
1B
7D
4D
6D
6D
2A
1D
2A
3B
4D
5D

D

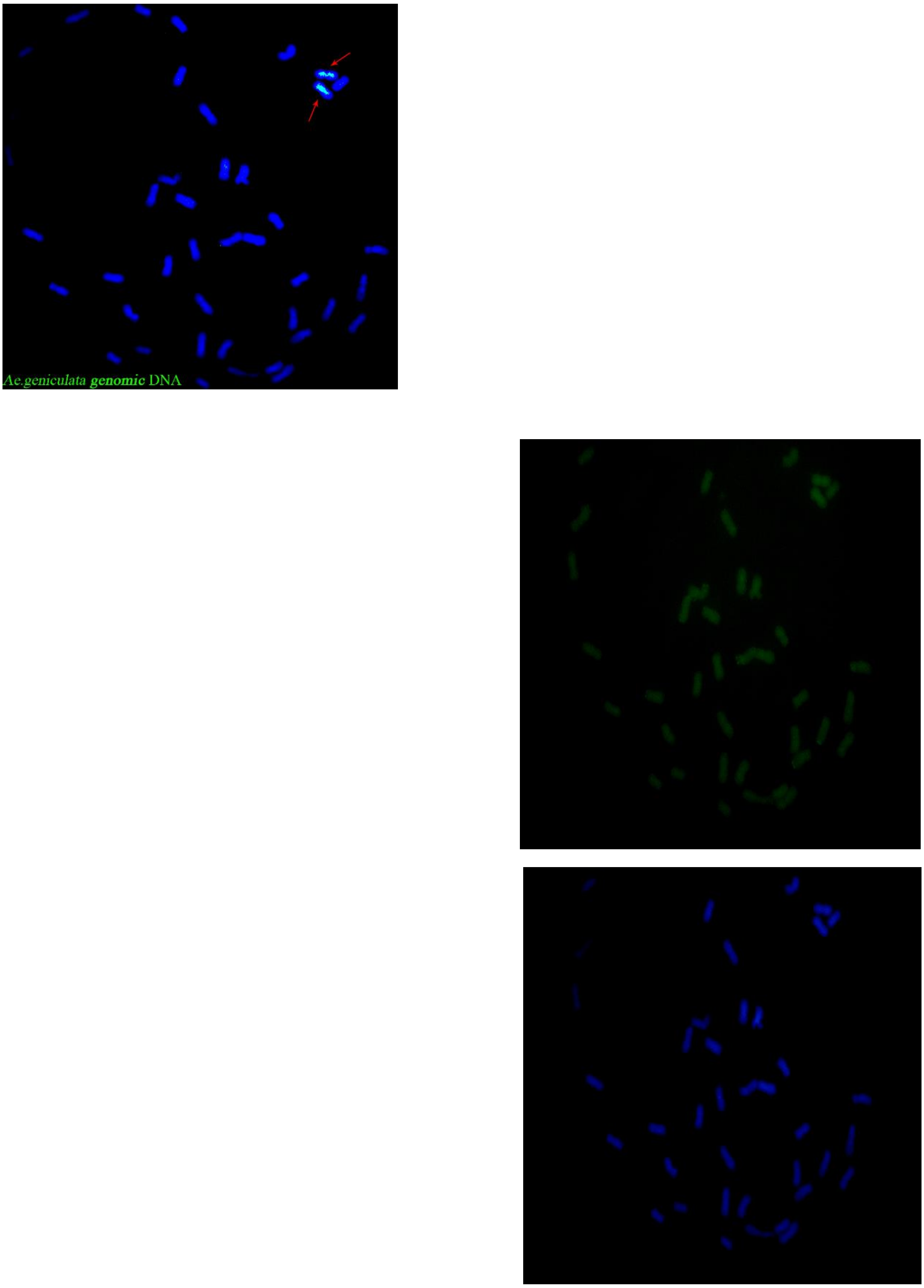

E

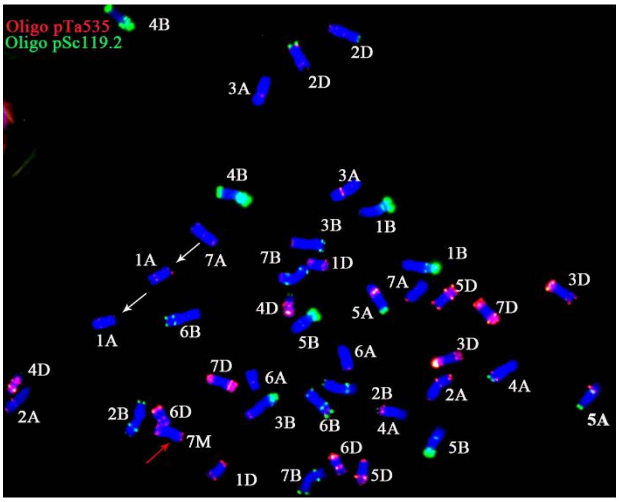
Oligo pTa535
Oligo pSc119.2
4B
2D
2D
3A
4B
3A
3B
1B
1A
7A
7B
1D
1B
7A
5D
3D
1A
6B
4D
5A
7D
5B
6A
3D
4D
7D
6A
2B
2A
4A
2A
2B
6D
3B
6B
4A
5A
7M
5B
6D
1D
7B
5D

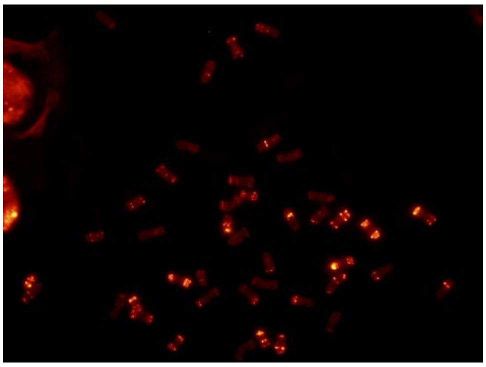

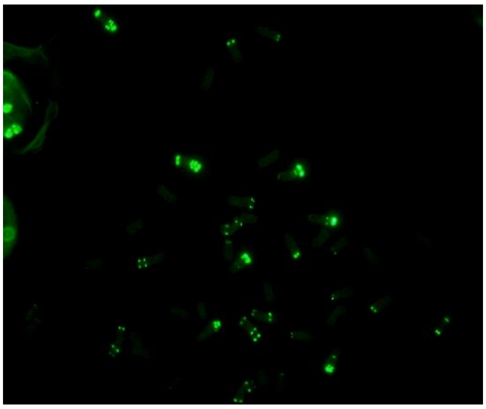

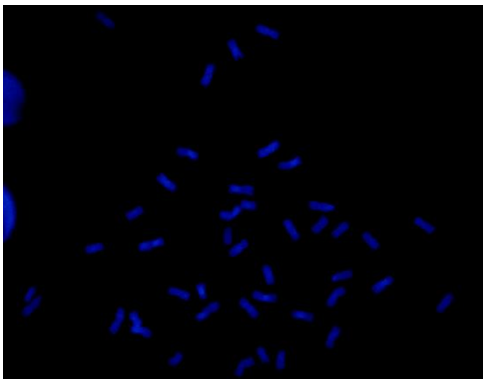

F

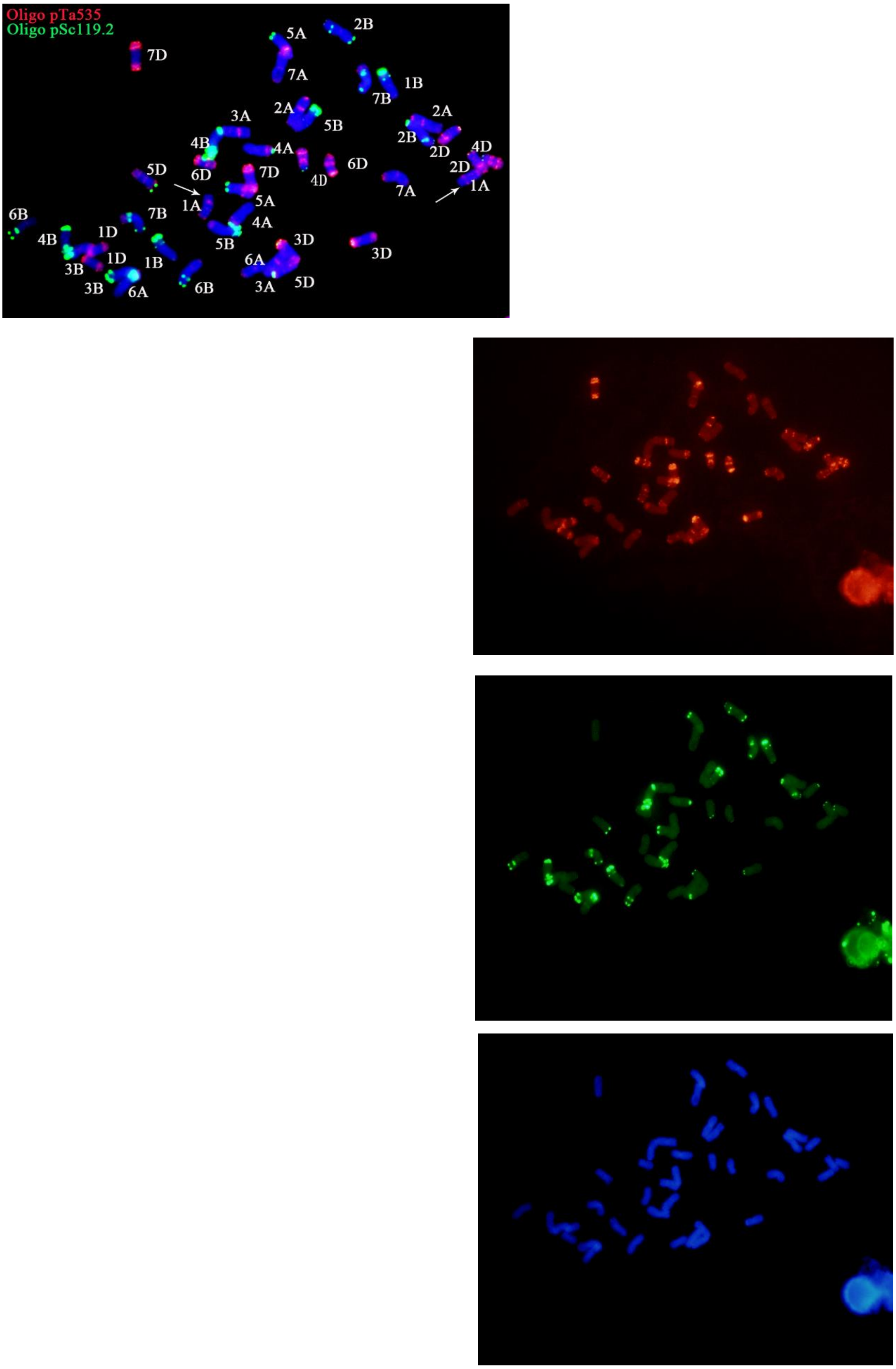
Oligo pTa535
Oligo pSc119.2
7D
5A
2B
7A
1B
7B
3A
2A
5B
2A
2B
2D
4D
4B
4A
2D
5D
6D
7D
4D
6D
7A
1A
1A
5A
6B
7B
4A
4B
1D
5B
3D
3D
3B
1D
1B
6A
3B
6A
6B
3A
5D

G

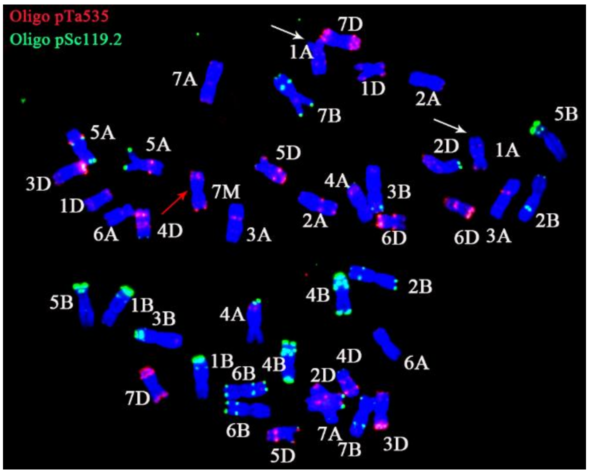
Oligo pTa535
Oligo pSc119.2
7D
1A
7A
1D
2A
7B
5B
5A
5A
5D
2D
1A
3D
1D
7M
4A
3B
2A
6A
4D
3A
6D
6D 3A
2B
5B
1B
3B
4A
4B
2B
1B
6B
4B
4D
6A
2D
7D
6B
7A
7B
3D
5D

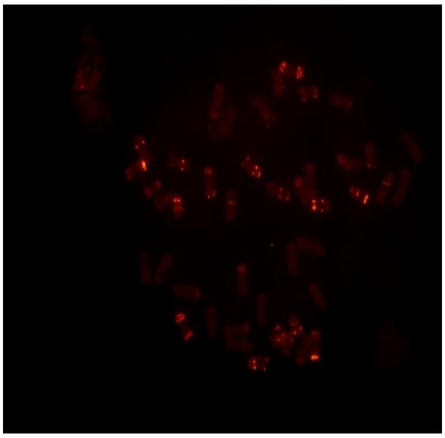

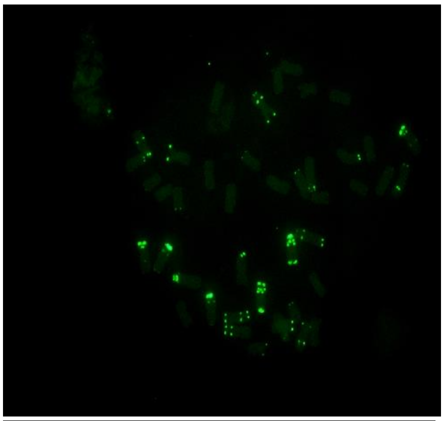

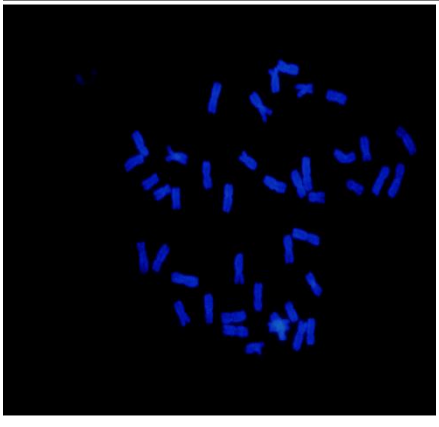

H

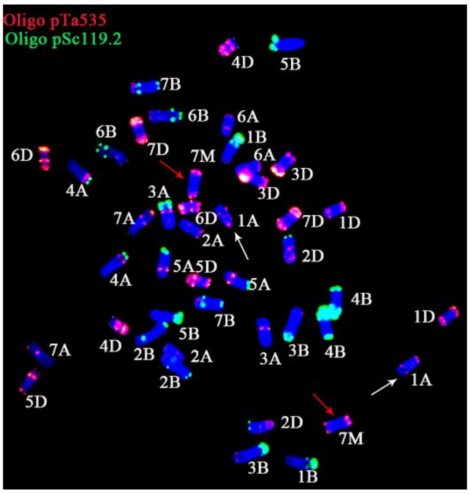

Oligo pTa535
Oligo pSc119.2
4D
5B
7B
6B
6A
1B
6B
7D
7M
6A
3D
6D
4A
3A
3D
7A
6D
1A
7D
1D
2A
2D
4A
5A5D
5A
4B
1D
7B
4D
5B
2B
2A
3A
3B
4B
7A
2B
1A
5D
2D
7M
3B
1B

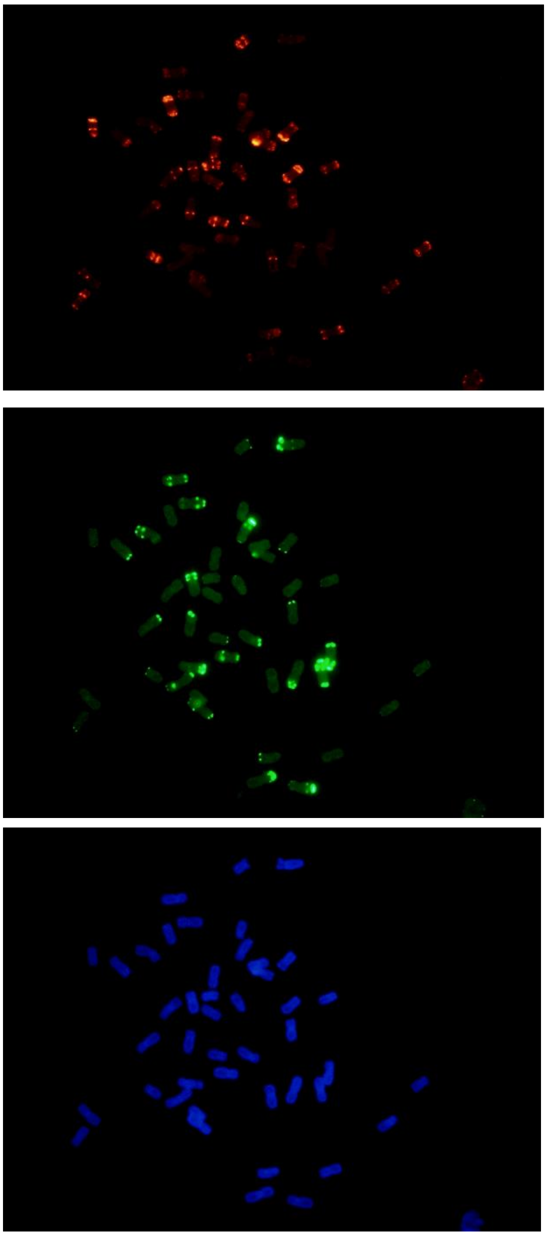

Figure S2. Uncropped images of Fig. 2.

The karyotype of the target plant was analyzed by FISH.

A

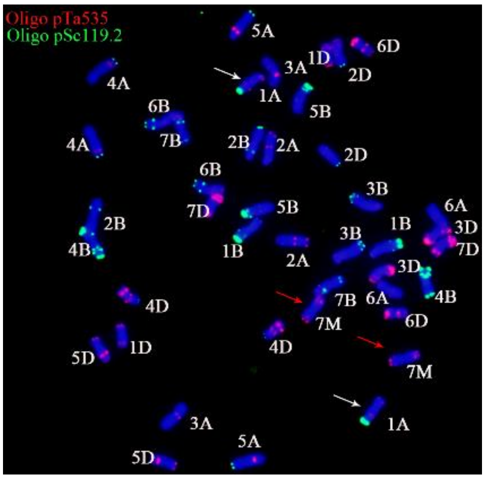

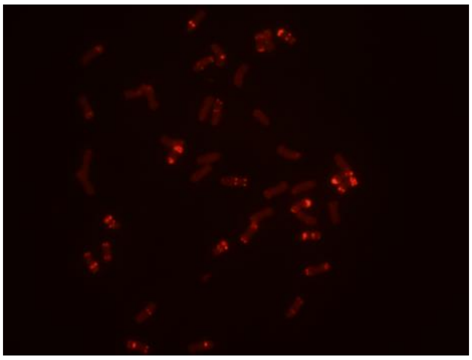

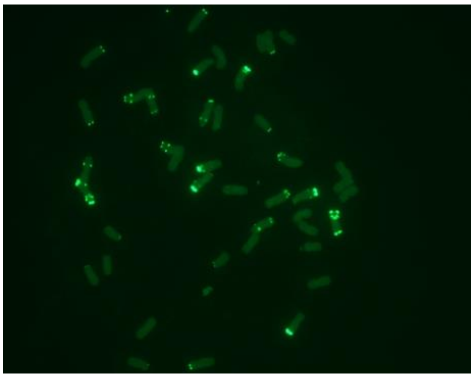

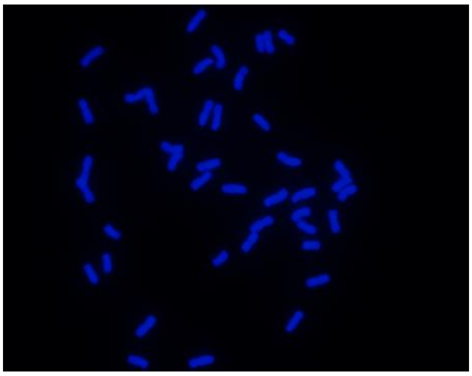

B

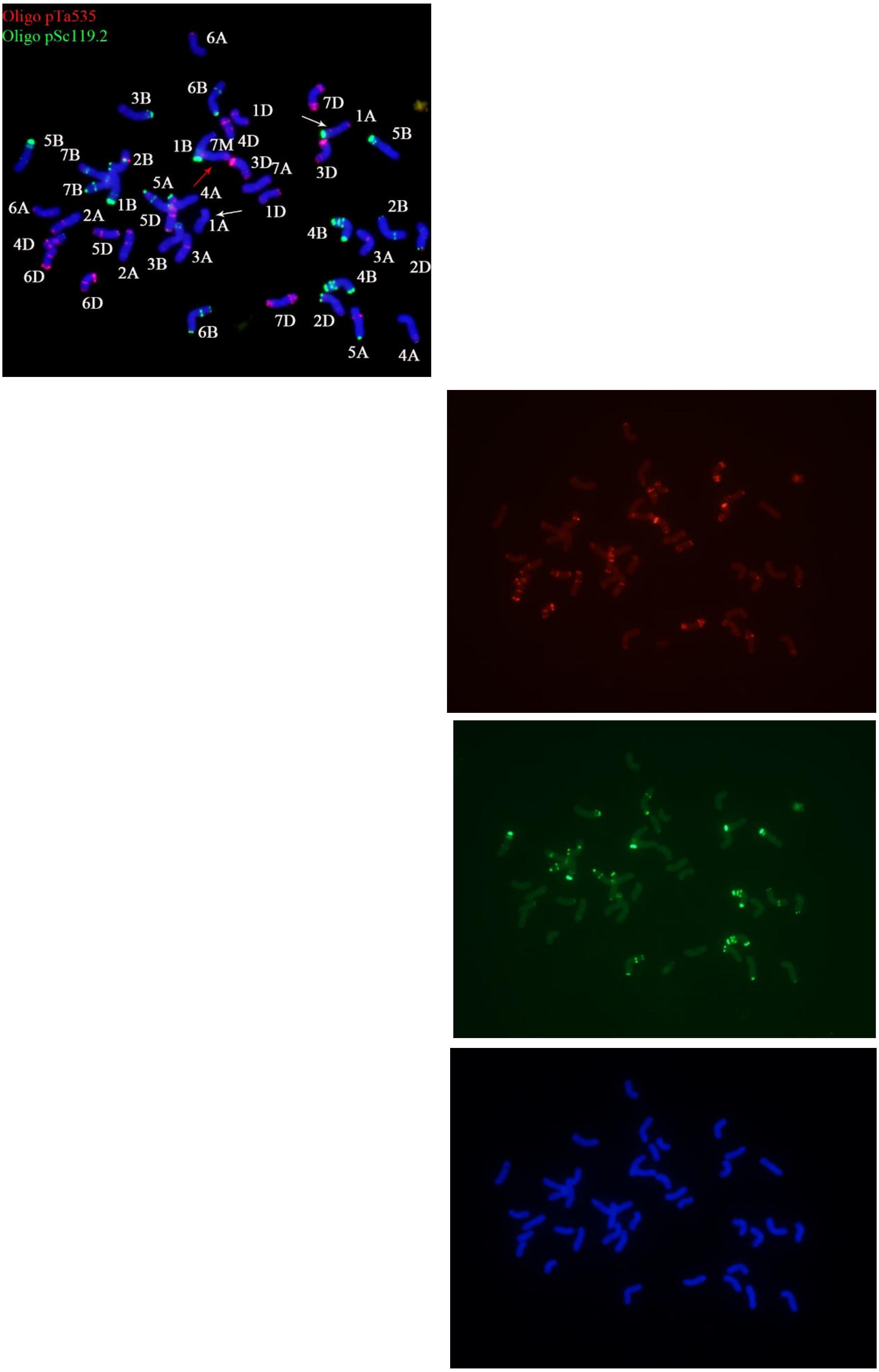
Oligo pTa535
Oligo pSc119.2
6A
6B
3B
1D
7D
1A
5B
5B
7B
2B
1B
7M
4D
3D
7A
3D
7B
5A
4A
6A
1B
2A
5D
1A
1D
2B
4D
5D
4B
3A
2D
6D
2A
3B
3A
4B
6D
7D
2D
6B
5A
4A

C

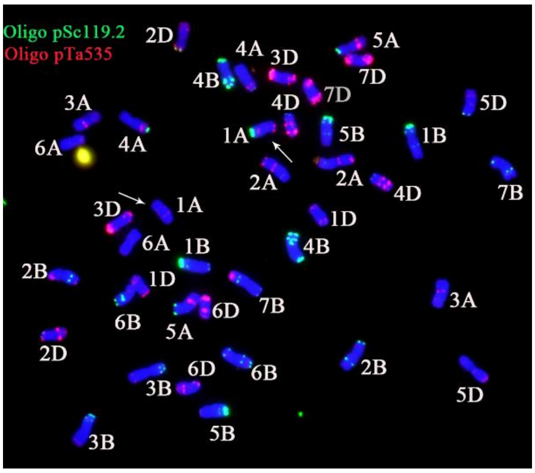

Oligo pSc119.2
Oligo pTa535
2D
4A
3D
5A
4B
7D
7D
4D
3A
5D
1A
5B
1B
6A
4A
2A
2A
4D
7B
3D
1A
1D
6A
1B
4B
2B
1D
6D
7B
3A
6B
5A
2D
6D
6B
2B
3B
5D
5B
3B

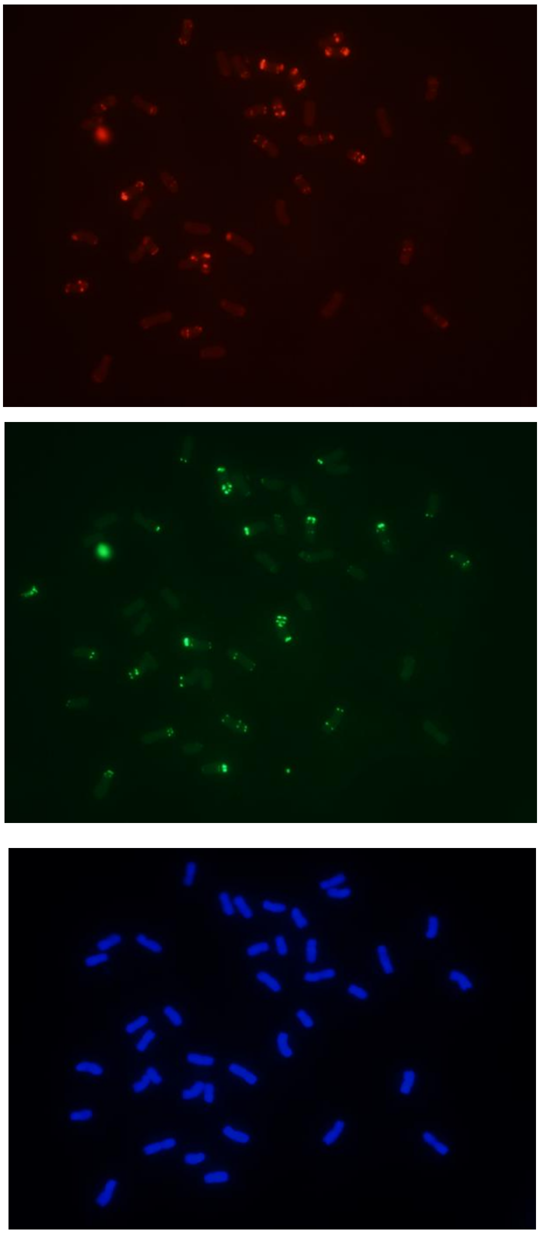

D

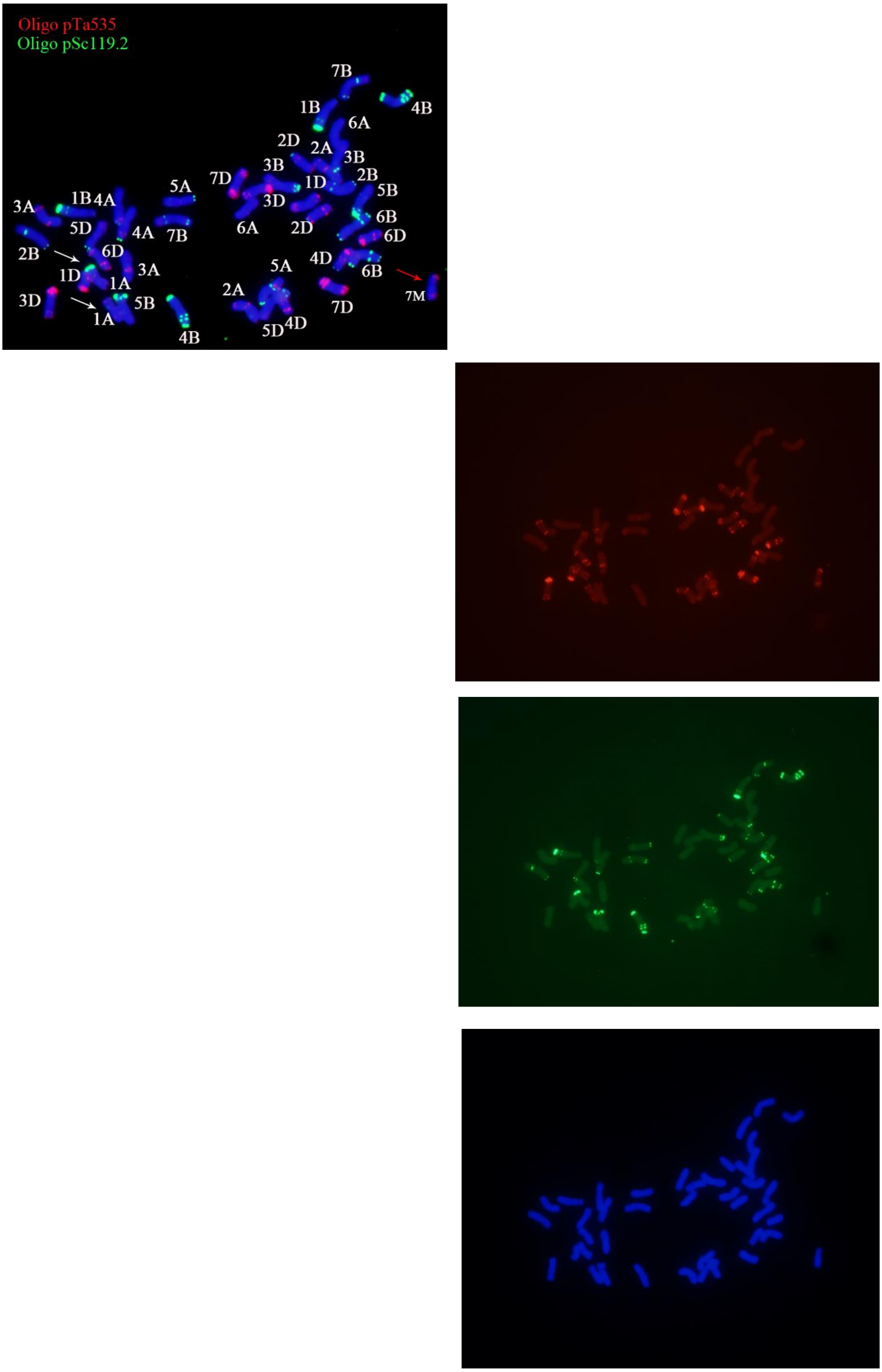
Oligo pTa535
Oligo pSc119.2
7B
4B
1B
6A
2D
2A
3B
3B
2B
7D
1D
5B
5A
3D
3A
1B
4A
6B
6D
5D
4A
7B
6A
2D
2B
6D
4D
6B
3A
1D
1A
5A
7M
3D
5B
2A
7D
1A
4B
5D
4D

E

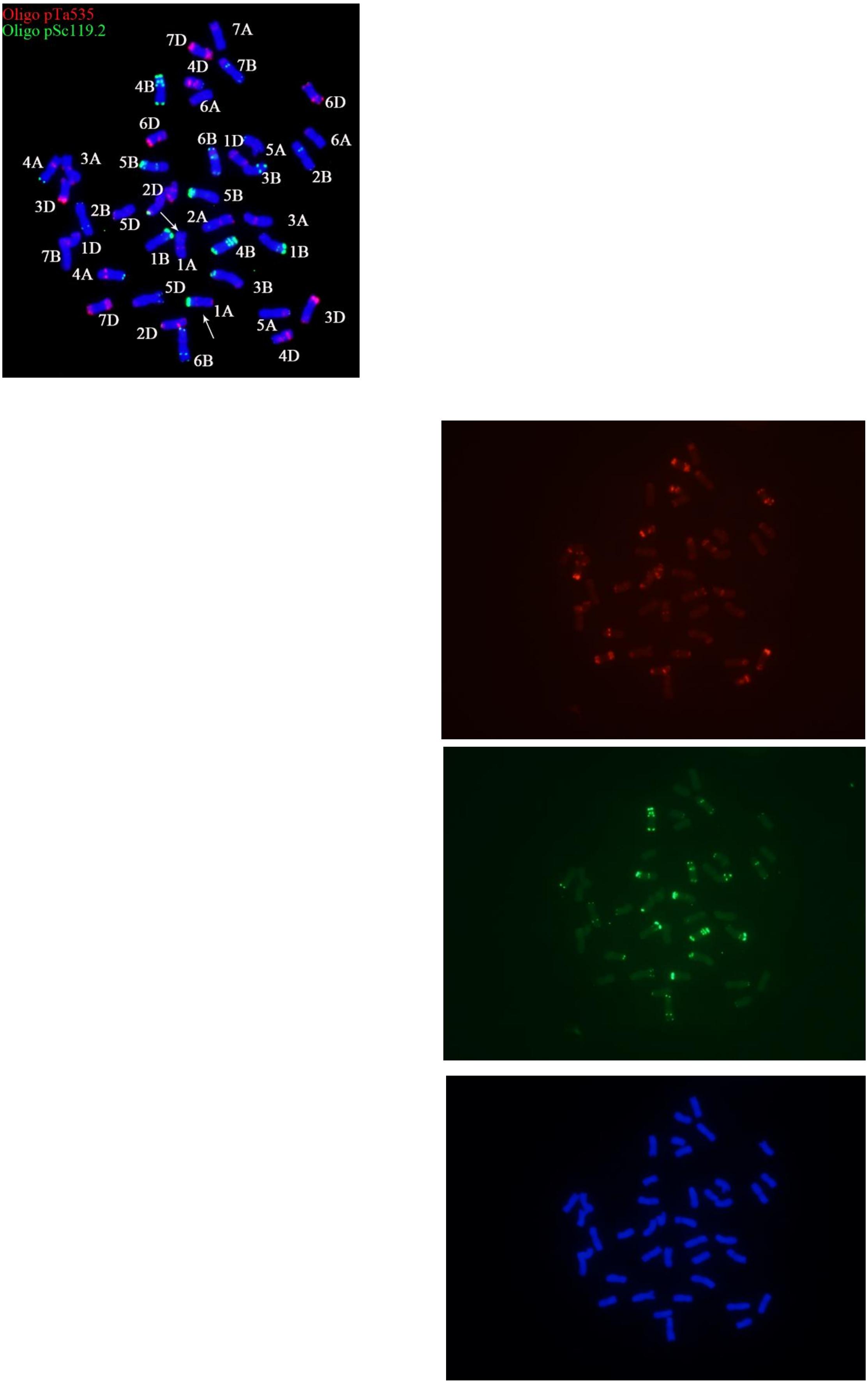
Oligo pTa535
Oligo pSc119.2
7A
7D
4D
7B
4B
6A
6D
6D
6B
1D
5A
6A
4A
3A
5B
3B
2B
2D
5B
3D
2B
5D
2A
3A
7B
1D
1B
1A
4B
1B
4A
5D
3B
1A
7D
2D
5A
3D
6B
4D

F

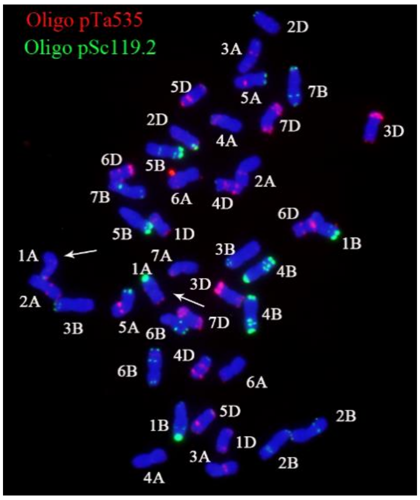

Oligo pTa535
Oligo pSc119.2
2D
3A
5D
5A
7B
7D
3D
2D
4A
6D
5B
2A
7B
6A
4D
6D
5B
1D
1B
1A
7A
3B
1A
4B
3D
2A
4B
3B
5A
6B
7D
4D
6B
6A
5D
2B
1B
1D
3A
2B
4A

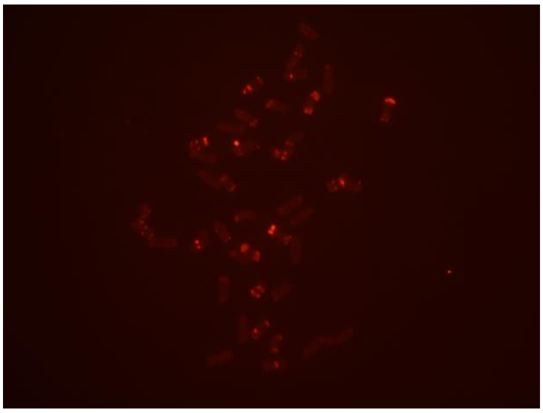

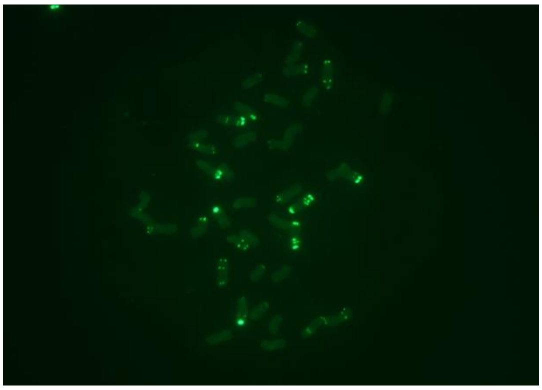

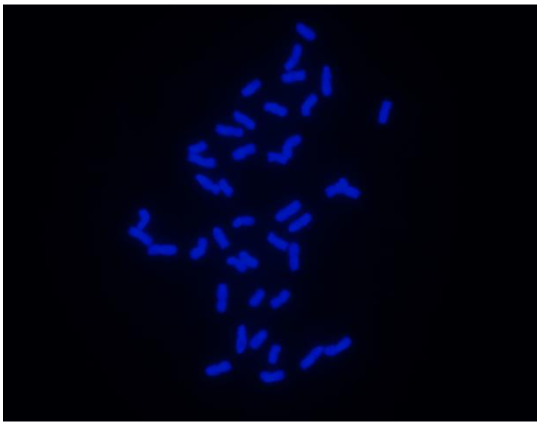

G

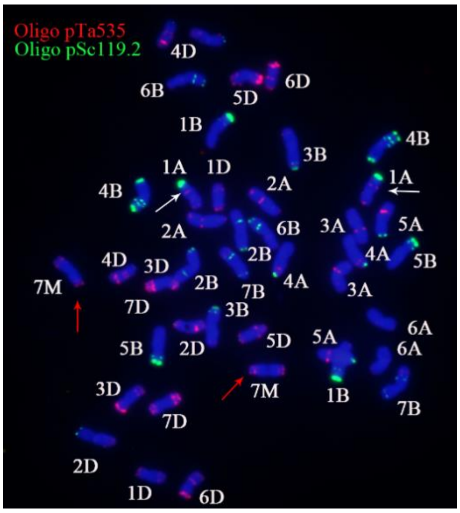
Oligo pTa535
Oligo pSc119.2
4D
6B
5D
6D
1B
3B
4B
1A
1D
2A
1A
4B
2A
6B
3A
5A
4D
3D
2B
4A
5B
7M
2B
7B
4A
3A
7D
3B
6A
5B
2D
5D
5A
6A
7M
1B
3D
7B
7D
2D
1D
6D

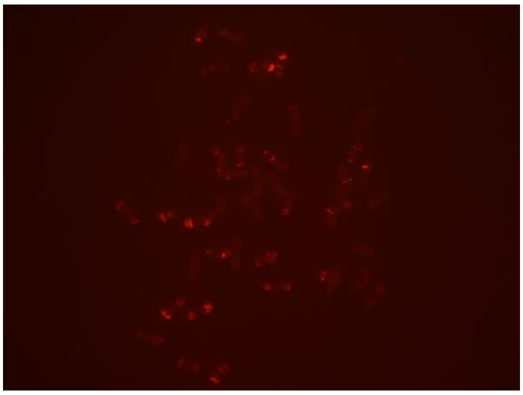

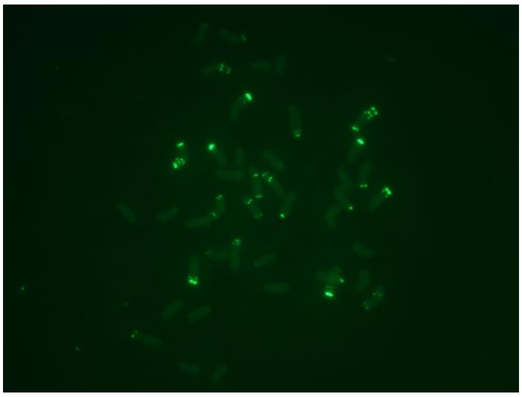

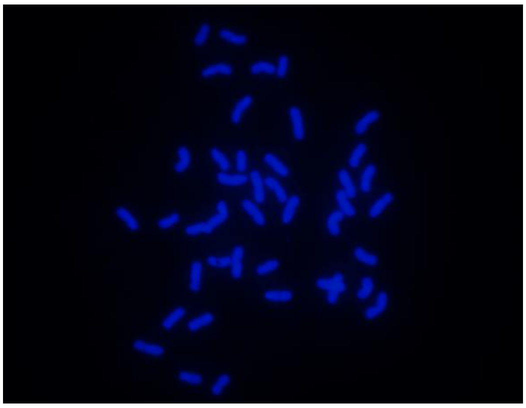

H

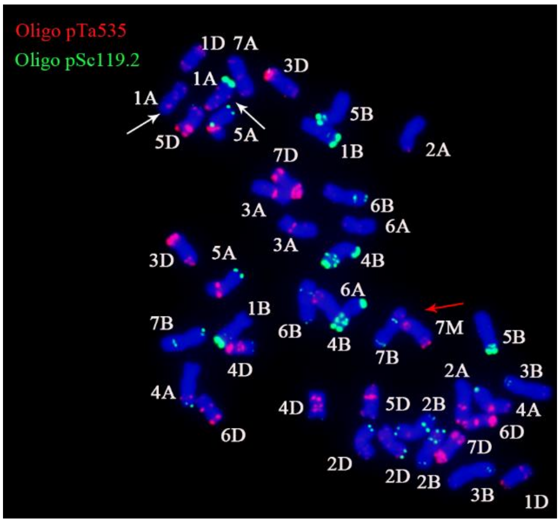
Oligo pTa535
Oligo pSc119.2
1D 7A
3D
1A
1A
5B
5D
5A
1B
2A
7D
3A
6B
6A
3A
3D
5A
4B
6A
1B
7B
7M
5B
6B
4B
7B
4D
2A
3B
4A
4D
5D
2B
4A
6D
6D
7D
2D
2D
2B
3B
1D

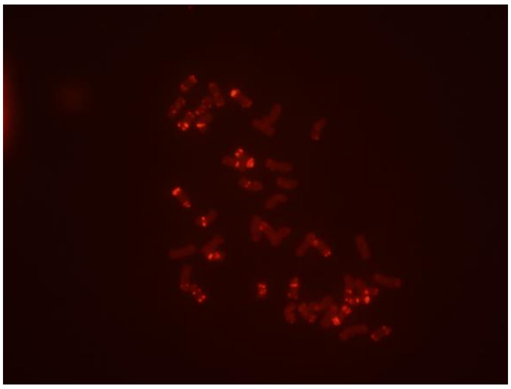

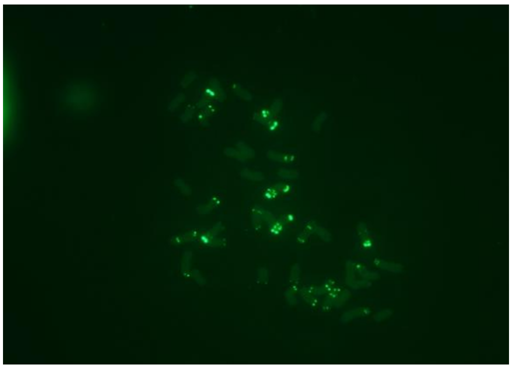

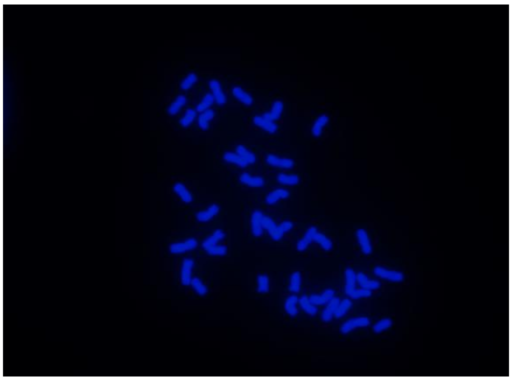

I

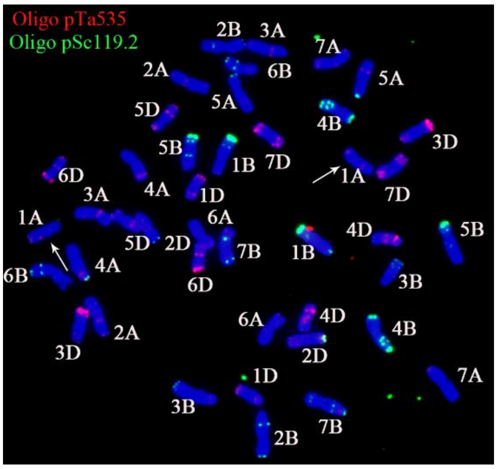
Oligo pTa535
Oligo pSc119.2
2B
3A
7A
5A
2A
6B
5A
5D
4B
3D
5B
1B
7D
1A
6D
4A
1D
7D
3A
6A
1A
5D
2D
7B
1B
4D
5B
6B
4A
6D
3B
2A
6A
4D
4B
3D
2D
1D
7A
3B
7B
2B

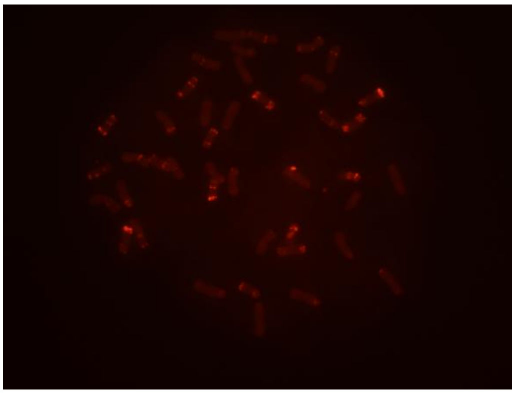

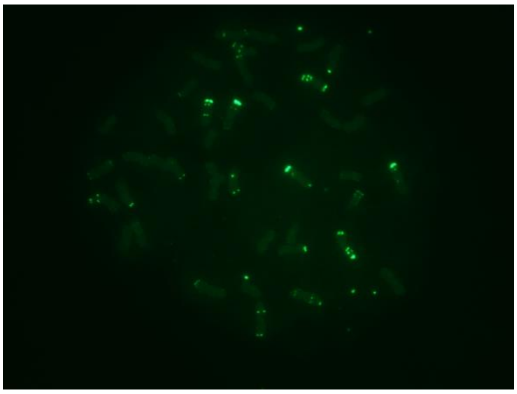

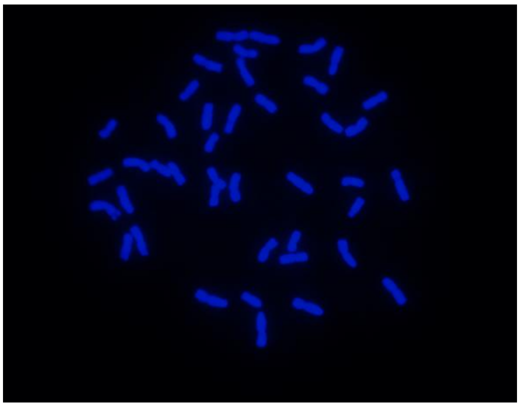

J

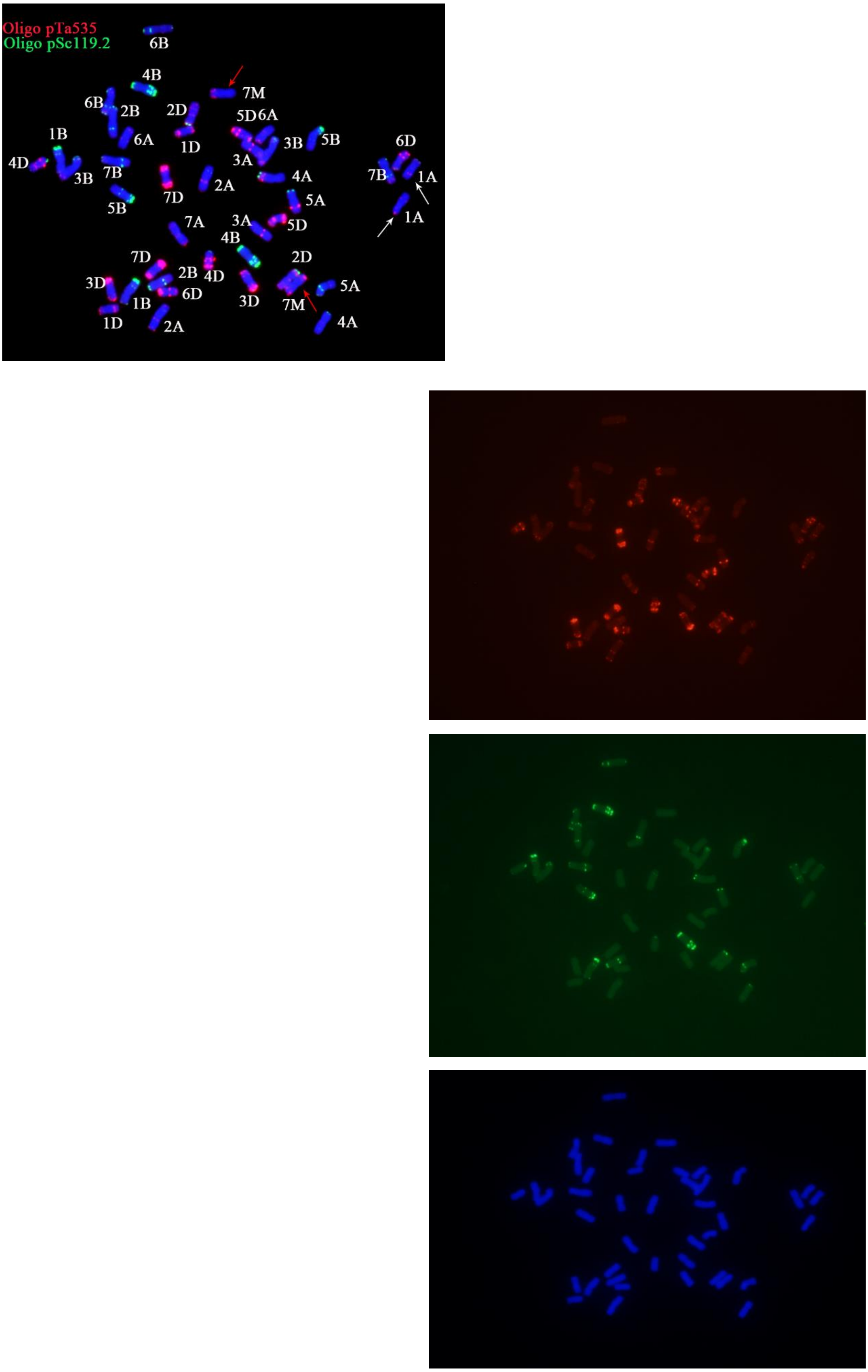
Oligo pTa535
Oligo pSc119.2
6B
4B
7M
6B
2B
2D
5D6A
1B
6A
1D
3B
5B
6D
4D
3B
7B
3A
4A
7B
1A
5B
7D
2A
5A
1A
7A
3A
5D
4B
7D
2B
4D
2D
3D
1B
6D
3D
7M
5A
1D
2A
4A

K

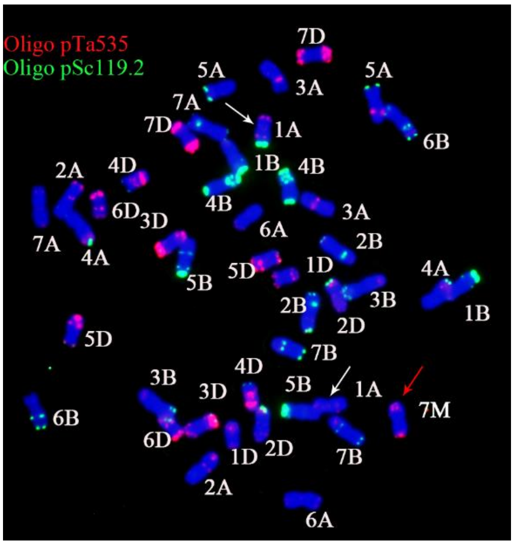
Oligo pTa535
Oligo pSc119.2
7D
5A
3A
5A
7A
1A
7D
6B
2A
4D
1B
4B
4B
6D
3D
3A
6A
7A
4A
2B
5D
1D
5B
4A
2B
3B
1B
2D
5D
7B
4D
3B
3D
5B
1A
7M
6B
6D
1D
2D
7B
2A
6A

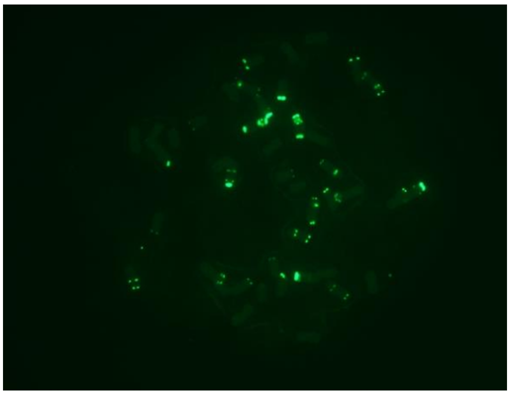

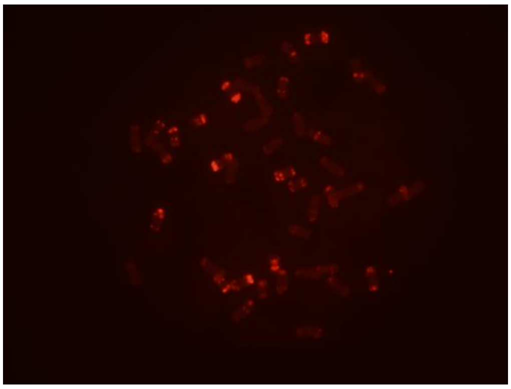

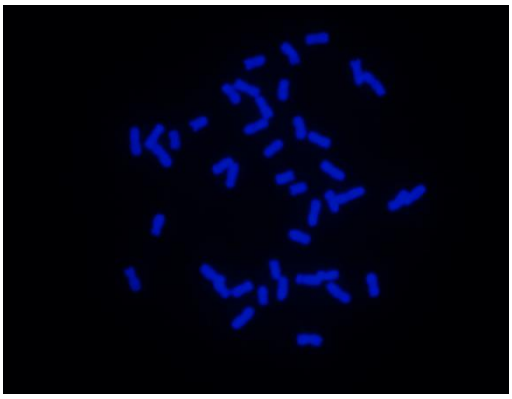

L

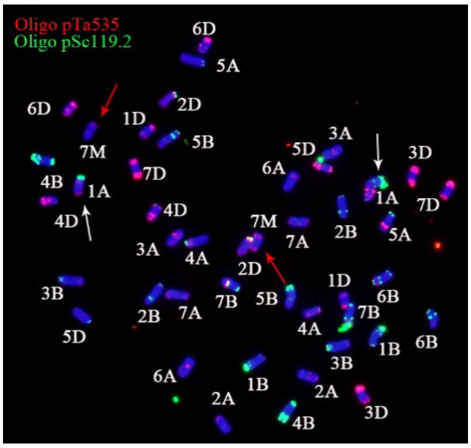
Oligo pTa535
Oligo pSc119.2
6D
5A
6D
2D
1D
5B
7M
3A
5D
3D
6A
7D
4B
1A
1A
7D
4D
4D
7M
2B
5A
3A
4A
7A
2D
1D
3B
7B
5B
6B
2B
7A
4A
7B
5D
1B
6B
3B
6A
1B
2A
2A
4B
3D

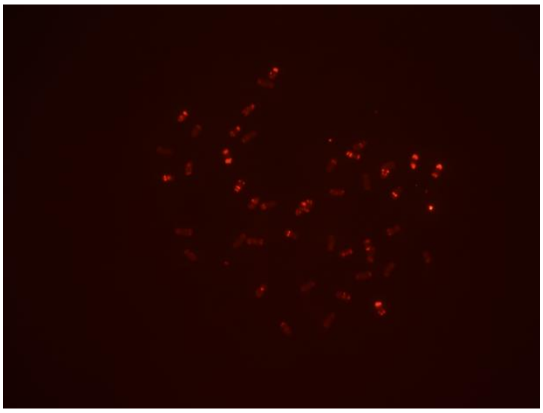

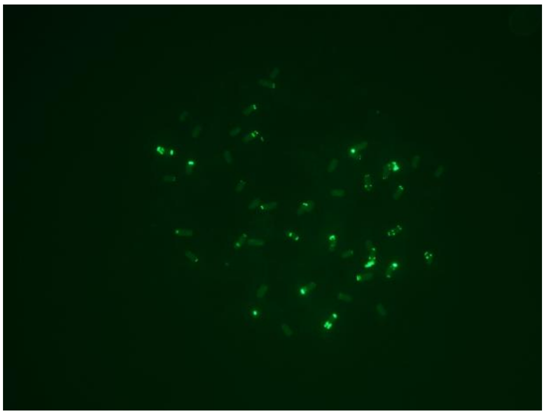

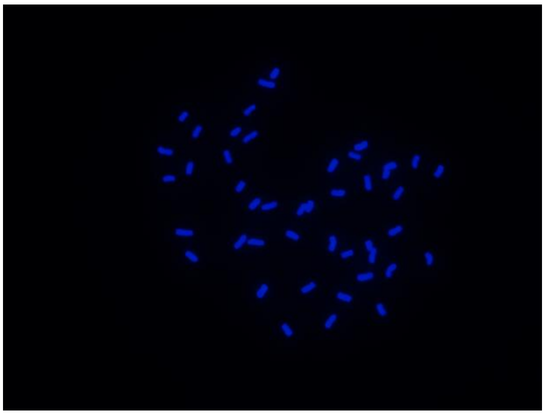

Figure S3. Uncropped gel images of markers of Fig. 3.

The EST–STS markers amplification results with *BE637663* (Fig. C-D).

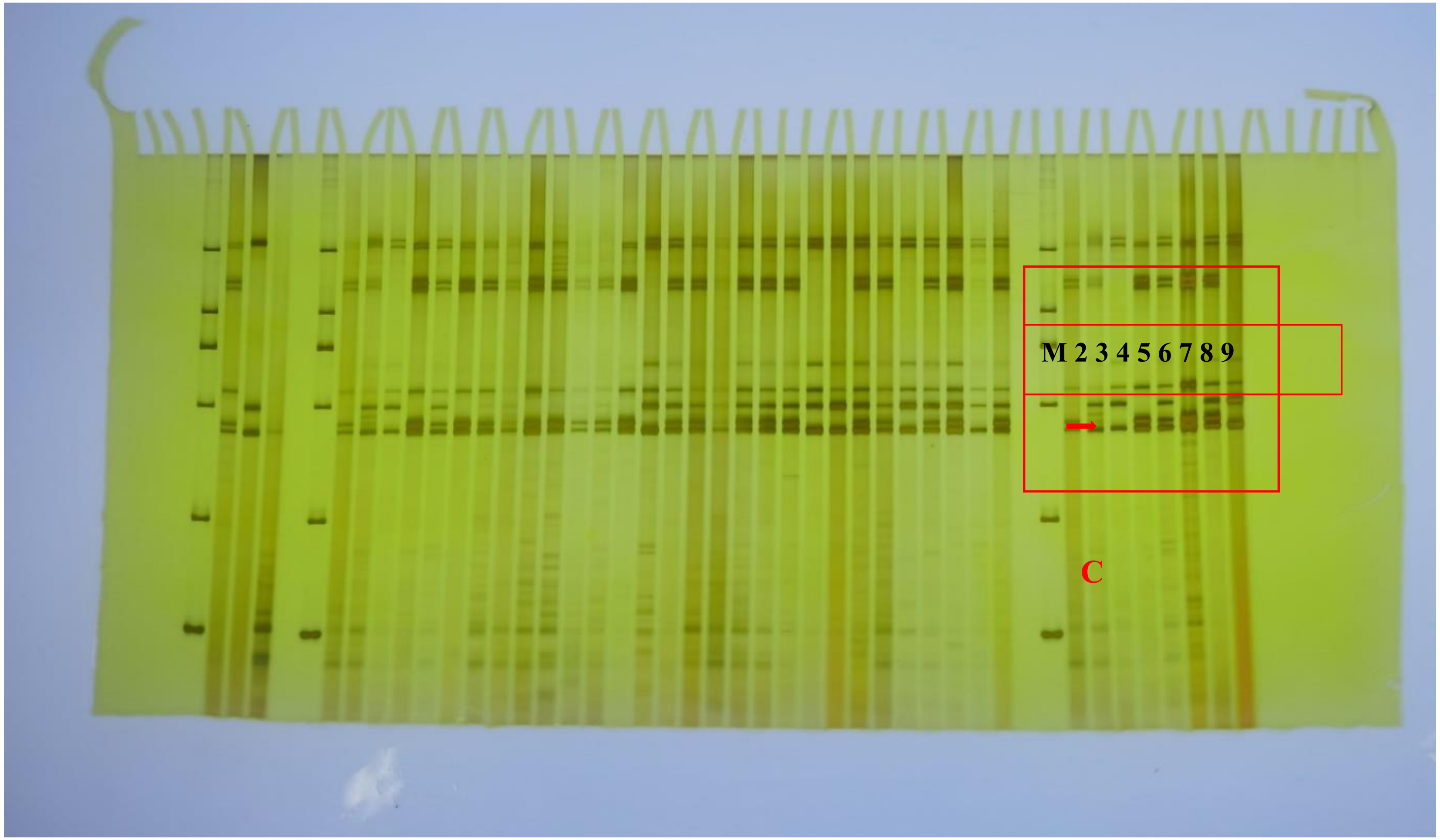

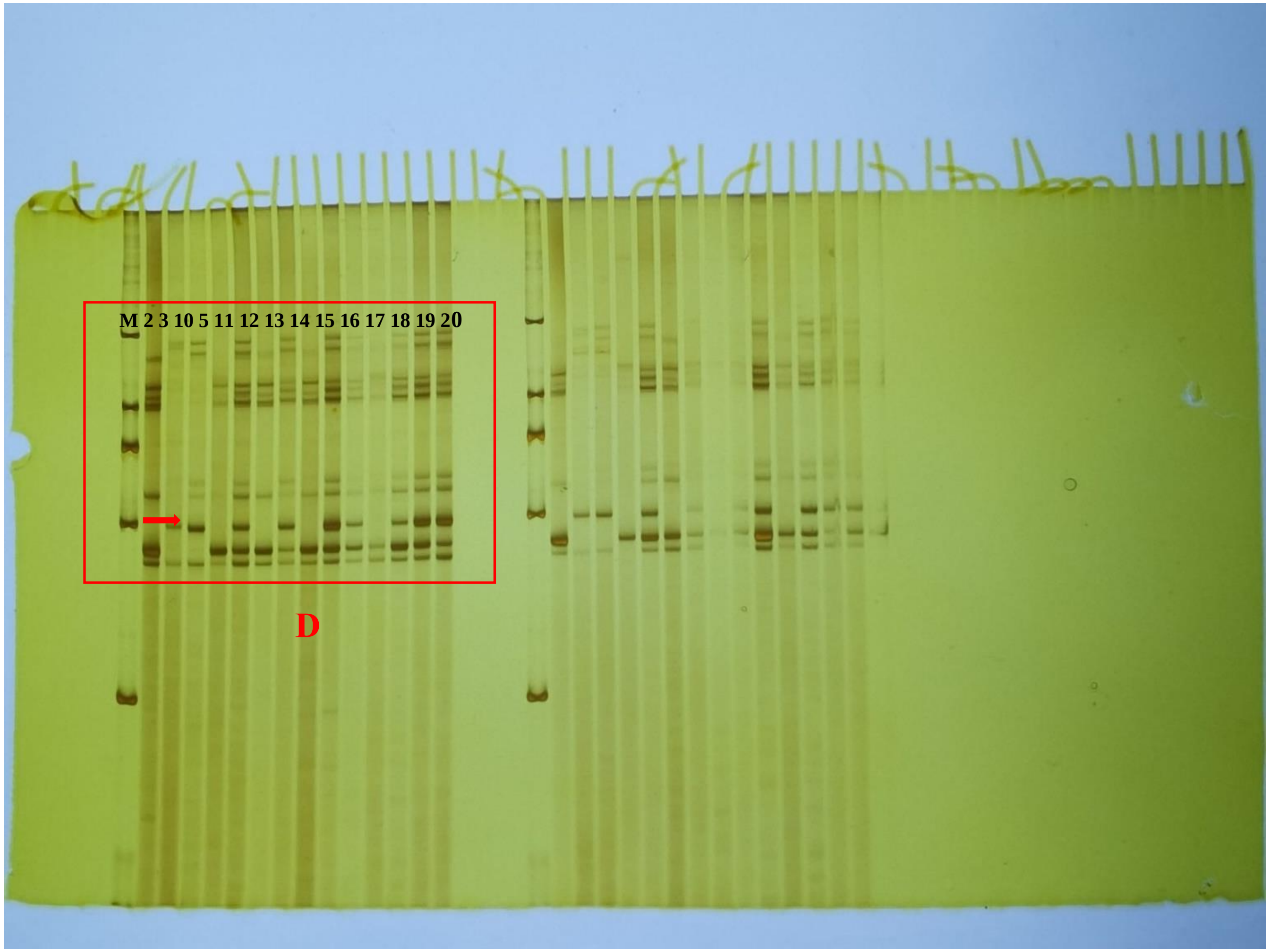
M 2 3 10 5 11 12 13 14 15 16 17 18 19 20
D

Figure S4. Uncropped gel images of markers of Fig. 4.

Amplification patterns of Aegilops accessions using NA0973-5-4-1-2-9-1 and W16998. SLAF markers *Marker7*, *Marker9*, *Marker65*, *Marker945*, *Marker1661*, *Marker2024*, *Marker887*, Marker2104 and Marker390. (M) DL2000 (2 kb DNA ladder). (1). CS. (2) SY159. (3). NA0973-5-4-1-2-9-1. (4). W16998. The red arrows indicate the specific bands.

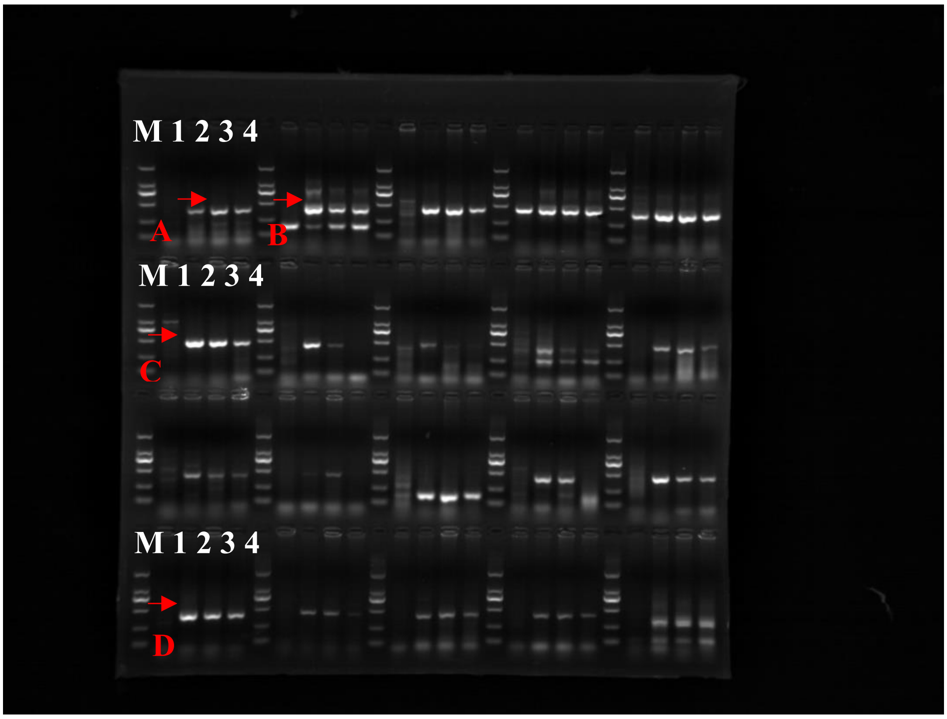

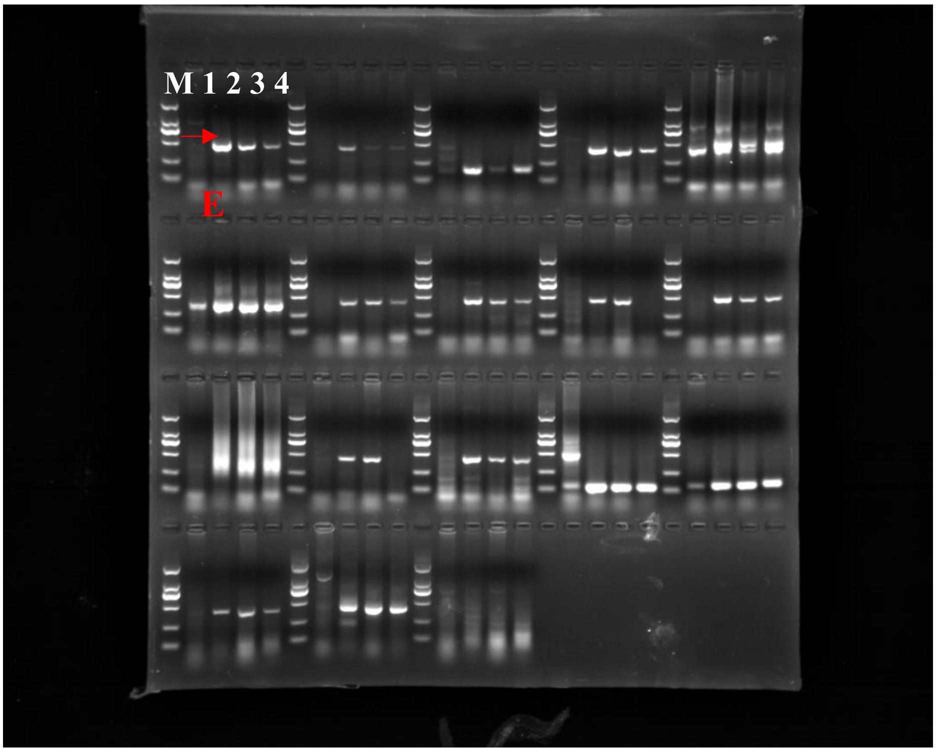

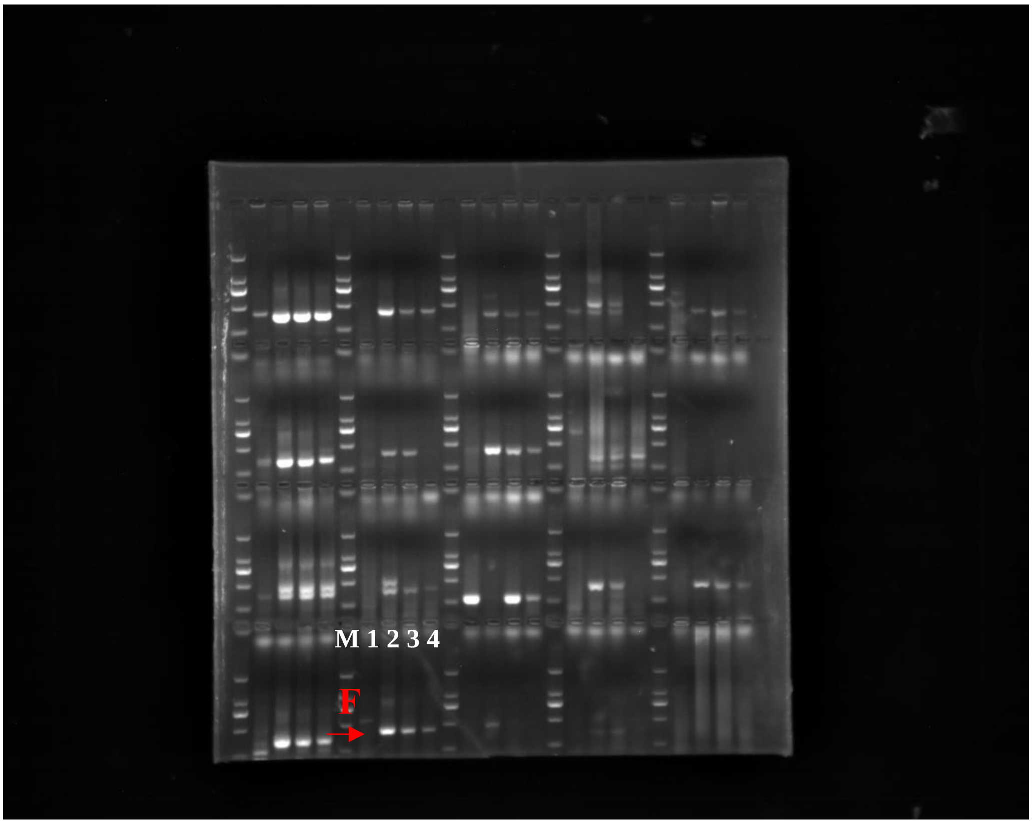
M 1 2 3 4
F

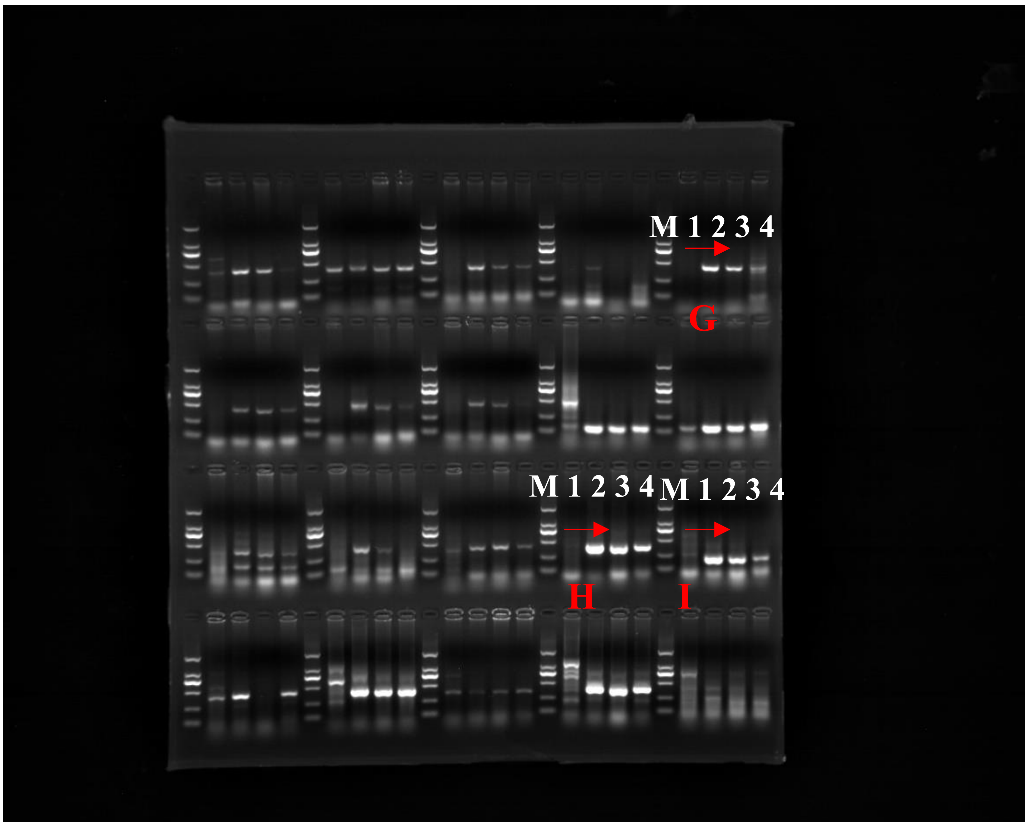
M 1 2 3 4
G
M 1 2 3 4
M 1 2 3 4
H
I

Figure S5. Uncropped gel images of markers of Fig. 5.

Molecular marker development and PCR amplification in target material.

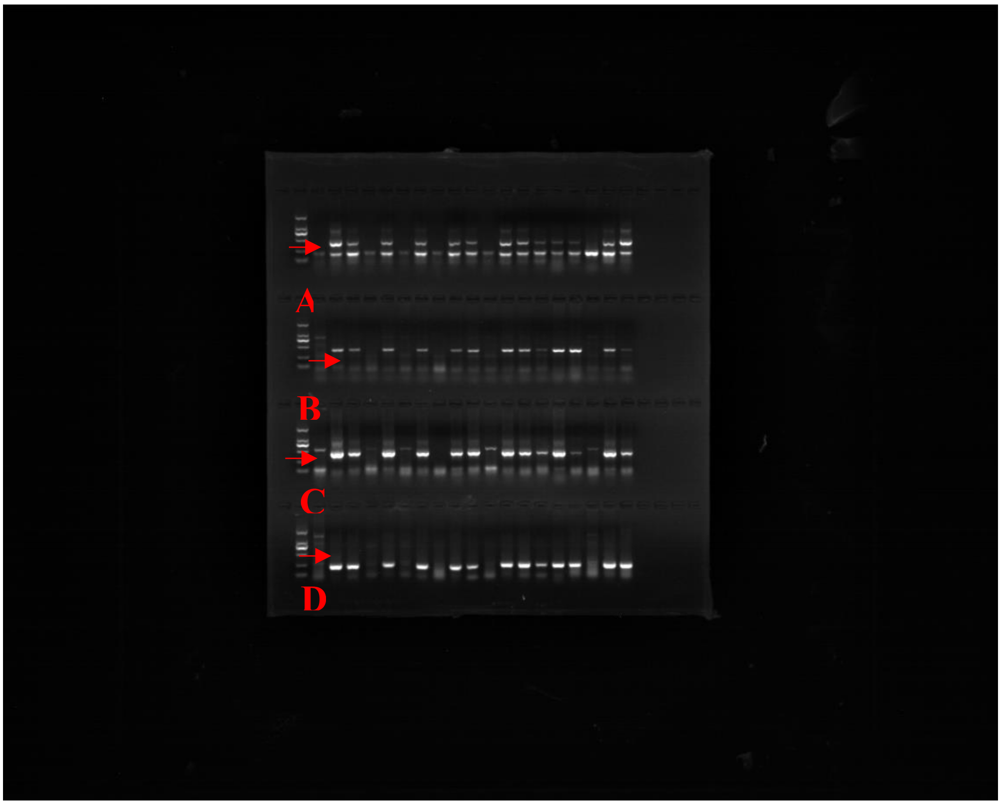

TableS2. Special SLAF-seq markers list 7M[g].

Number	#Gene_ID	RPr1(5'-3')
1	Marker7-F	GCTACACCGAACGACAATCA
2	Marker7-R	AGGTGTCCGCTAAGGATTGA
3	Marker9-F	CAGACGAGCTTGACTGCTTG
4	Marker9-R	AATTTGTGCCGATTCAAAGG
5	Marker37-F	TCGTTTGTTCACATGGTCGT
6	Marker37-R	AACTGGGTGGAGATGATTCG
7	Marker56-F	TTGATCACTGTACCCTCCTTCA
8	Marker56-R	TGGTGGCATATCAATCCAGA
9	Marker27-F	ACATTGGGGGTATCAAACCA
10	Marker27-R	TGTTGACACGGATGAAGCTC
11	Marker65-F	GCTGCAGCAAAGCATTTACA
12	Marker65-R	AGCACGTCCAAAAGAGGCTA
13	Marker44-F	TGACTGTATCCCTCGATGCT
14	Marker44-R	GACCTAGCGCTCTGGATGTC
15	Marker415-F	ACTACTGAGGAAAGGCGCAG
16	Marker415-R	ATGTCGAGCCCTTTGACATC
17	Marker55-F	TCCGTAGAGCCGGAACTAAA
18	Marker55-R	TCCAATTTAGGCAGCATCAT
19	Marker1783-F	CTGGGTCGACTTGTGATGTG
20	Marker1783-R	TACGTGTGTTTGTTCGTCGG
21	Marker1465-F	AGCAGATCACCCAGAGCAGT
22	Marker1465-R	CCTCCTATGCATTGCCTTTT
23	Marker75-F	TCATCCTCTCTCTCAGGGGA
24	Marker75-R	TCCTCCCCTTTTTCCTTGTT
25	Marker113-F	GAGTCATCCGAAGGAATGGA
26	Marker113-R	TGATTTTCTTCATGAGGGGG
27	Marker2298-F	TGCTTGGTGAATAGACTGCG
28	Marker2298-R	TGAGAAGAGCAGCAAGTCCA
29	Marker106-F	GGTTCGTGGGACTTGAGTGT
30	Marker106-R	GGGAGGGGTTTGTGTCATAG
31	Marker945-F	CTTCCGAGCTCCGGTAAGAT
32	Marker945-R	TGATGTGGTGATCCTTTCCA
33	Marker774-F	CCAGTGTCAGGTTGATCCAG
34	Marker774-R	TTTGGTGCGAAGTTTGTTTG
35	Marker1423-F	GGGGATCTAGCAGTTGGGTT
36	Marker1423-R	GGTGCATCCGATTTATCCAC
37	Marker1521-F	GGGCTTGGGCATTACTAACA
38	Marker1521-R	TCCTAACTGGACCTTTCCCA

39	Marker2224-F	GGTCCCATAGGTGTCGTCC
40	Marker2224-R	GCAGCTGCACATATCGCTAA
41	Marker753-F	CCCAACTCCACAGAGGTTGT
42	Marker753-R	CCCCGGGTCACTTTTCTAAT
43	Marker422-F	CAATGAATTGCCTTTTTGGG
44	Marker422-R	TTGTAGGGCGCAGAAAAGAT
45	Marker1568-F	AGCCCGCTTCTCTTCTCTCT
46	Marker1568-R	ACCGTGGCAGATGTCGATAC
47	Marker403-F	GGCTTCTCATTCCACAGTCC
48	Marker403-R	AAGTCTTGCCGTTGTTGCAT
49	Marker579-F	ACCTTAGTCTGCGCAGTGGT
50	Marker579-R	CAAACCAAGCATCCCTGAAT
51	Marker1975-F	TACGCAATCAAGGACACCAC
52	Marker1975-R	TATGTGTTACAGGGGTGCGA
53	Marker1661-F	TTGGCATACAAGCCAACTCA
54	Marker1661-R	AGAGTGCTCTGGTCGGAAAG
55	Marker161-F	GTTGTGCAAAGAGTTGTGCG
56	Marker161-R	ACGGATTCAAACATGCCACT
57	Marker1757-F	TGTACAAAGTCTTGCCGCTG
58	Marker1757-R	GCCCCTCTTCTCCTCTCTGT
59	Marker1842-F	ACTGCGGCAACTTTACACCT
60	Marker1842-R	TATGGTGTGGCTATGCTCCA
61	Marker338-F	GGCTTCTCATTCCACAGTCC
62	Marker338-R	ATCCTTCGTACAGAGCCAGG
63	Marker925-F	TGCCAACTTACAACACGACA
64	Marker925-R	TCTCAAAGGATGGCAAGGAC
65	Marker1608-F	GAAGTGGAACTTCTCGGCAG
66	Marker1608-R	CGCCTTCGATTACTCCTCTG
67	Marker125-F	GCATTGATGGTCTGATGGAA
68	Marker125-R	TGCCTAACTTGAGCAGCAGA
69	Marker1944-F	GGAAACGTTCTTCTTGAGGGT
70	Marker1944-R	GCCCTCCTCAAAATCCTCTT
71	Marker1250-F	CCTTCAGGAGCGACAACTTC
72	Marker1250-R	AATGTTTGTCCTAAAGCGGG
73	Marker2024-F	CTTTGGTCCCTTCTTTGGGT
74	Marker2024-R	GCTTAATAGATGCCGAAGCG
75	Marker1451-F	ATGGGAAGTTGCAGATCGTC
76	Marker1451-R	TCTCGCCTATATTGCGTTCC
77	Marker121-F	GAGTGGCTTTAGGTCTCCCA
78	Marker121-R	ATGGGTTCCACTCGACAGAC
79	Marker314-F	TGCAGCTCGCTTAGTTGTTG
80	Marker314-R	GCACTTTGTCATCGAGCCAT
81	Marker1153-F	TTTTCGCGGTACTCAAATCC

82	Marker1153-R	TTCGGGATGCATGGAGTAAT
83	Marker2295-F	TCCCCAGCCTCAATGAATAG
84	Marker2295-R	TTTCCTGCAAGCAAACAATG
85	Marker1790-F	GAGAGGCGTACCACATGGAT
86	Marker1790-R	TCTGGCACACAAACTTCGTC
87	Marker1864-F	AGTTCGTCACCCACACCATC
88	Marker1864-R	ATTCATCGTCTTGGCAGGAG
89	Marker887-F	GCATGGGTCCTGTCGACTAT
90	Marker887-R	TCAGTGGCTGAAGGTCAGTG
91	Marker432-F	GACAAGGGGTTTCACTCCAA
92	Marker432-R	TACACATGGCGAAGCTGATG
93	Marker93-F	AGGCAGCTGTAATACGCCAT
94	Marker93-R	GCAGCCCCTTTAGTTGTTGA
95	Marker2070-F	GGACCAAAGTTGTGGCTCAT
96	Marker2070-R	TATTGAAAATGGGGCAAAGG
97	Marker2135-F	GAGAAGGGGTAGGAAGGCAC
98	Marker2135-R	GTCAAGGGCACGACAAAAGT
99	Marker1821-F	GCCTTCTCTTGAGGGAGCTT
100	Marker1821-R	ACAAACCACCAGCGGTAAAC
101	Marker2369-F	GGACGATGCATGTTGTTGTC
102	Marker2369-R	TACACACGCAAGTCCCAGAG
103	Marker2081-F	CATCGTACCTCTCGCAACAA
104	Marker2081-R	ACATTGCCGGTAGAAGATGG
105	Marker1832-F	TTGTAGCTCCAGTGGGGAAC
106	Marker1832-R	TCTATCCGAGCTGGAAGCAT
107	Marker2104-F	GCCCAAGGATGAGATGCTAA
108	Marker2104-R	AGAAGAGGGGAAACTCGACC
109	Marker390-F	GGAAGTTTACCGAAGATGGC
110	Marker390-R	GGGAGCTAAAGAAAGCCGAT
111	Marker891-F	TGAAGGGTCTCATCTCGCTT
112	Marker891-R	TTGTTCAAGGAAAACCAGGG
113	Marker488-F	GTGGCGATTCCTGTCATTTT
114	Marker488-R	TCACAGAAGTCCGAGTGGTG
115	Marker860-F	ATATTGCACAATGGGGGAAA
116	Marker860-R	GCCACTGGTGTTCCTCCTAA
117	Marker1459-F	AATGAAAAGGGGGTGTTTGC
118	Marker1459-R	GGAGGCTTCAAGGACGAAG
119	Marker2143-F	GCTAGCCACTCTGGATGGAG
120	Marker2143-R	CTATTCAAGGGAACGCGAAG
121	Marker1588-F	GCCAGGCTTCAATGTTTTGT
122	Marker1588-R	ACATGTGACCTGTCCTGCTG
123	Marker587-F	GGCGGAGCTACAGAAAAAGTT
124	Marker587-R	AGATTAGGGTGGCGATTCCT

125	Marker1670-F	GAGCTGGAGCAGTCGAAGAC
126	Marker1670-R	GACATGAACGGATTCTCGGT
127	Marker781-F	CACAAAGATCTGCATGCCAC
128	Marker781-R	ATCATCAGAGGATCTTGGCG
129	Marker531-F	GTACGAATCGGAACAGGGAA
130	Marker531-R	TGCAAGATACTCCCTCGGTT
131	Marker1425-F	CAATTTCTGTCACGCAAGGA
132	Marker1425-R	CGAATATGCGCATGCTGTAG
133	Marker1680-F	ATGTGCCAGGTTTCACATCA
134	Marker1680-R	GGGGGTTTCTTCACCTCAAT
135	Marker2077-F	GTGTTACCCTAAGCCGGTCA
136	Marker2077-R	TCTTCCCCCTTTCCTCAGAT
137	Marker1651-F	GATCCATAGCCATCCACCAC
138	Marker1651-R	TTAAGAAAGCATCACCCGCT
139	Marker809-F	TCAGCTTTTCAATCGATCCC
140	Marker809-R	TAGCCCTACGTCTCGAGTGC
141	Marker1283-F	GCACCTTTCTGATGCCTTTC
142	Marker1283-R	AGAGCGATGTTAAAGCGGAA
143	Marker397-F	GCACGCAGCTCAACAAGATA
144	Marker397-R	TGTACTGTTTCCTTGGCGTTT
145	Marker784-F	CTCACACTCGATGGCAACAT
146	Marker784-R	TCCGCCTGAGAGAGAGAGAG
147	Marker653-F	CTCATTGACCGACACAATGG
148	Marker653-R	AAATTGGTCGACTCTGCACC
149	Marker2263-F	CTAACCAGTGGCGAAACCAT
150	Marker2263-R	TCGAGAGCGATCAACCTCTT
151	Marker2373-F	CGATACTCACTTGATGGGCA
152	Marker2373-R	AAGACCCGGATCAAAAGGAT
153	Marker1374-F	AGCAGTACCACATTTTGGGC
154	Marker1374-R	CCCTCGAAAAGCCTTGCTAT
155	Marker1800-F	TCGAATATGCAAAAGAAATAAGCA
156	Marker1800-R	AAGCAGCAGCCAGAGAAGAG
157	Marker2186-F	TTTACTAGCCGCATGATCCC
158	Marker2186-R	TATTTCTAAGCCGGAGCTGG
159	Marker2091-F	TGTTGGGTAATGGGTTGGAT
160	Marker2091-R	TCCAGAACTTATCATCCGCC
161	Marker2212-F	AACCCGATCCAGCGATTAG
162	Marker2212-R	TCATGCAAAGATCCGAAGAA
163	Marker561-F	GTTCCTGGTGTCGTTGCTCT
164	Marker561-R	AAGCGACACGTGTTAAATGC
165	Marker955-F	CACGTGAAAGTCACGGAGAA
166	Marker955-R	CACCTGCCGTCTACACTCAA
167	Marker1038-F	GTTGGACGGCATAGACTTGG

168	Marker1038-R	AGACTTGCAAAAGGGAAGCA
169	Marker1311-F	CAAAGACCATGTGGCTGATG
170	Marker1311-R	GGTCATGGTTTGGCACTCTT
171	Marker303-F	TTGTGAAAATCTCATTGCCAG
172	Marker303-R	GCAGTAGCTCGATCCTGTCC
173	Marker1601-F	GACTTGCTACGTCAGTTGCG
174	Marker1601-R	CCCATTCATCTATTGCATCG
175	Marker2297-F	GATTGGGTATGCCCGTTATG
176	Marker2297-R	TGAAGCACCAACCAATGAAA
177	Marker769-F	TCCAGATCTTCCTCCCTTCA
178	Marker769-R	GCCTAAGAAGAGAACCACCCA
179	Marker1170-F	CTTCGTGGCATTTGGACTTT
180	Marker1170-R	AGACCTCGACCTCCAAGGAT
181	Marker1506-F	ATAGGTGTCGTTCCCCGAC
182	Marker1506-R	ATGGCTTCATGCAAAGATCC
183	Marker1619-F	GCTATCTTCGCTTTTGTGGG
184	Marker1619-R	CCAAACATGTCGGGAAGAGT
185	Marker1943-F	AATCCCGATCACATCCTGAG
186	Marker1943-R	AGCTGGAGGCATGATCAAAT
187	Marker1652-F	CGTGGAGTCTGAAAAGAGGG
188	Marker1652-R	CCTCCCTGAAAGGCTTTACC
189	Marker1688-F	GCGGAGGTTTTCTGAGATTG
190	Marker1688-R	GAGGTCGTGGGAGGTTTCTT
191	Marker647-F	GTAAACCGACATGCCCACTC
192	Marker647-R	AGAATGGATCATCACCCTGC
193	Marker394-F	GCGCATCAGGAGTAACCTCT
194	Marker394-R	CGCGTCCGAGACAATCTT
195	Marker903-F	ATGAATGAGATGCTCCCAGC
196	Marker903-R	GCACGATACTTACGTGGCAA
197	Marker989-F	GATGTCTGCCTCTTCTTCCG
198	Marker989-R	CTTCGCCCTCGATAGGACTT
199	Marker1074-F	CACTTCAGAGGCAGGAGGAG
200	Marker1074-R	TATATAAGGGGCGAAAGGGG
201	Marker1178-F	CCCTTCAAGCTACCGCATAG
202	Marker1178-R	GGCTGAATCCAGCAGAAACT
203	Marker898-F	GCTTACTGTTCAAGAGCGGG
204	Marker898-R	TGGGAATTACCAAAAATGGC
205	Marker1318-F	CGGGTTTTCATAGTGGAGGA
206	Marker1318-R	GCCACACAACTTAGCCACAA
207	Marker1572-F	GCTCTGGGTGAGAATTCCATA
208	Marker1572-R	TCCTCTTCCCAATCTCCACT
209	Marker2195-F	CGAGCTCCTTGAGATATGCC
210	Marker2195-R	GGGACCTGAGTCTGAAGCTG

211	Marker144-F	CAACGGCTAGAACAGCATCA
212	Marker144-R	CATCTCCTCCTCGGTGTCAA
213	Marker1152-F	CATTTTCCAACCTAGCTCGC
214	Marker1152-R	GATCACACGGGCAGTCTTTT
215	Marker1394-F	TCATCAAATGTGCAAAGGAGA
216	Marker1394-R	CTCGGGAATGACACAGACCT
217	Marker2036F	GCGGAGGTTTTCTGAGATTG
218	Marker2036-R	AGGCGTGGGAGGTTTCTTT
219	Marker401-F	CCATAGTTGCCTAGGATTGTTG
220	Marker401-R	ACGGACAACCTGGTTAGGTG
221	Marker104-F	GCTAGACACTCGTCAAGGGC
222	Marker104-R	GCTGGCAAGACTCCTCAGAC
223	Marker1385-F	GCTCCTCCAAGCTGTCAAAG
224	Marker1385-R	CTAGGATCCAGCGTCTCAGG
225	Marker661-F	ATACACCCAAGACGCACACA
226	Marker661-R	ATGCCCACATGTTGTTCAAA
227	Marker1035-F	AGACAAGGCAGAGCTCCAAG
228	Marker1035-R	TAAACCGCCATATTGCCTTC
229	Marker197-F	TCTTTGGTCCCCTCTCTGG
230	Marker197-R	CAATTTGAAGCTTTGAGGGG
231	Marker1169-F	GGAGATCGAGGGTGGTTTCT
232	Marker1169-R	GGGTAGTCAAAACGGGTGG
233	Marker697-F	AAACATCTCAACCCTCCACG
234	Marker697-R	CATAAGCCCGCAAAATGACT
235	Marker1840	AACATCCCTTCCTCGTCCTC
236	Marker1840	GGGTGGGTTGATTTTTCTTG
237	Marker664-F	GCTCGGTCTTTAAGGCGTC
238	Marker664-R	GGATCGAGGGACGTTTGAG
239	Marker885-F	AGATGTCTCGAGCACGCA
240	Marker885-R	TGCAAAATTCATCTAAAGTTGAGC
241	Marker803-F	GCTTACTGTTCAAGAGCGGG
242	Marker803-R	AACATGGGCAGAGGTAACAGA
